# Supplementary material for: Single-cell transcriptomic analysis in a mouse model deciphers cell transition states in the multistep development of esophageal cancer
Source: Nat Commun. 2020 Jul 24;11:3715. doi: 10.1038/s41467-020-17492-y (PMC7381637; doi:10.1038/s41467-020-17492-y)
Supplement: Supplementary file 1 — Supplementary Information [file 41467_2020_17492_MOESM1_ESM.pdf]

Supplementary Information

Single-cell transcriptomic analysis in a mouse model deciphers cell transition states in the multistep development of esophageal cancer

Jiacheng Yao, Qionghua Cui, Wenyi Fan, Yuling Ma, Yamei Chen, Tianyuan Liu, Xiannian Zhang, Yiyi Xi, Chengcheng Wang, Linna Peng, Yingying Luo, Ai Lin, Wenjia Guo, Lin Lin, Yuan Lin, Wen Tan, Dongxin Lin*, Chen Wu* and Jianbin Wang*

*Correspondence:

jianbinwang@tsinghua.edu.cn
chenwu@cicams.ac.cn
lindx@cicams.ac.cn

This file includes:
Supplementary Figure 1 to 7
Supplementary Table 1 to 3

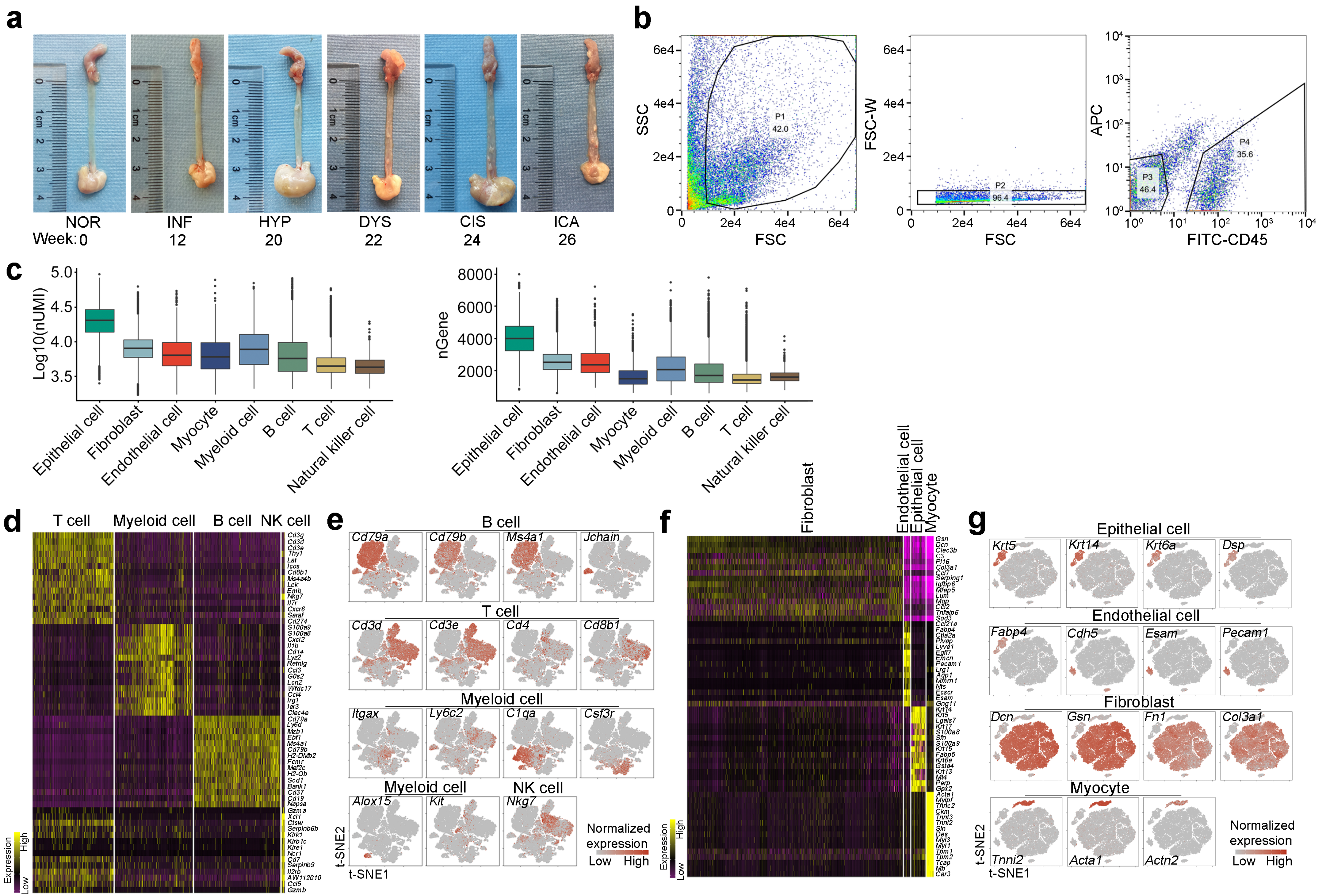
a
NOR
INF
HYP
DYS
CIS
ICA
Week: 0
12
20
22
24
26
b
SSC
FSC
P1
42.0
FSC-W
P2
96.4
APC
FITC-CD45
P3
46.4
P4
35.6
c
Log10(nUMI)
nGene
Epithelial cell
Fibroblast
Endothelial cell
Myocyte
Myeloid cell
B cell
T cell
Natural killer cell
d
T cell
Myeloid cell
B cell
NK cell
Expression
High
Low
e
B cell
Cd79a
Cd79b
Ms4a1
Jchain
T cell
Cd3d
Cd3e
Cd4
Cd8b1
Myeloid cell
Itgax
Ly6c2
C1qa
Csf3r
Myeloid cell
NK cell
Alox15
Kit
Nkg7
t-SNE2
t-SNE1
Normalized expression
Low
High
f
Fibroblast
Endothelial cell
Epithelial cell
Myocyte
Expression
High
Low
g
Epithelial cell
Krt5
Krt14
Krt6a
Dsp
Endothelial cell
Fabp4
Cdh5
Esam
Pecam1
Fibroblast
Dcn
Gsn
Fn1
Col3a1
Myocyte
Tnni2
Acta1
Actn2
t-SNE2
t-SNE1
Normalized expression
Low
High

Supplementary Fig. 1 Additional details in mouse model and marker gene expression across cell types. **a** Photos of longitudinally opened mouse esophagi. Gross anatomy of representative esophagi from 4NQO-treated mice showing gradually increased tumor burden. The scale in cm is shown on the left of the esophagus sample. **b** Pseudocolor image showing gating strategy of flow cytometry and cell sorting. P1 and P2 were to exclude cellular debris and doublets, respectively. P3 and P4 were to sort cells by FITC-labeled CD45 antibody. Color indicates dot density and number indicates percentage of cells. **c** Boxplots showing the number of unique molecular identifier (UMI) (left panel) and genes (right panel) in different types of cells, colored by cell type (center line, median; box limits, upper and lower quartiles; whiskers, 1.5x interquartile range; points, outliers). Cell number was n = 1,756 for epithelial cells, n = 31,654 for fibroblasts, n = 1,197 for endothelial cells, n = 1,507 for myocytes, n = 9,465 for myeloid cells, n = 10,337 for B cells, n = 9,833 for T cells, n = 340 for NK cells. **d** Heatmap of marker gene expression of CD45+ cells including T cells, myeloid cells, B cells and NK cells. **e** tSNE of scale normalized expression of selected marker genes from (**b**) across cell types. **f** Heatmap of marker gene expression of CD45– cells including fibroblasts, endothelial cells, epithelial cells and myocytes. **g** tSNE of scale normalized expression of selected marker genes from (**d**) across cell types.

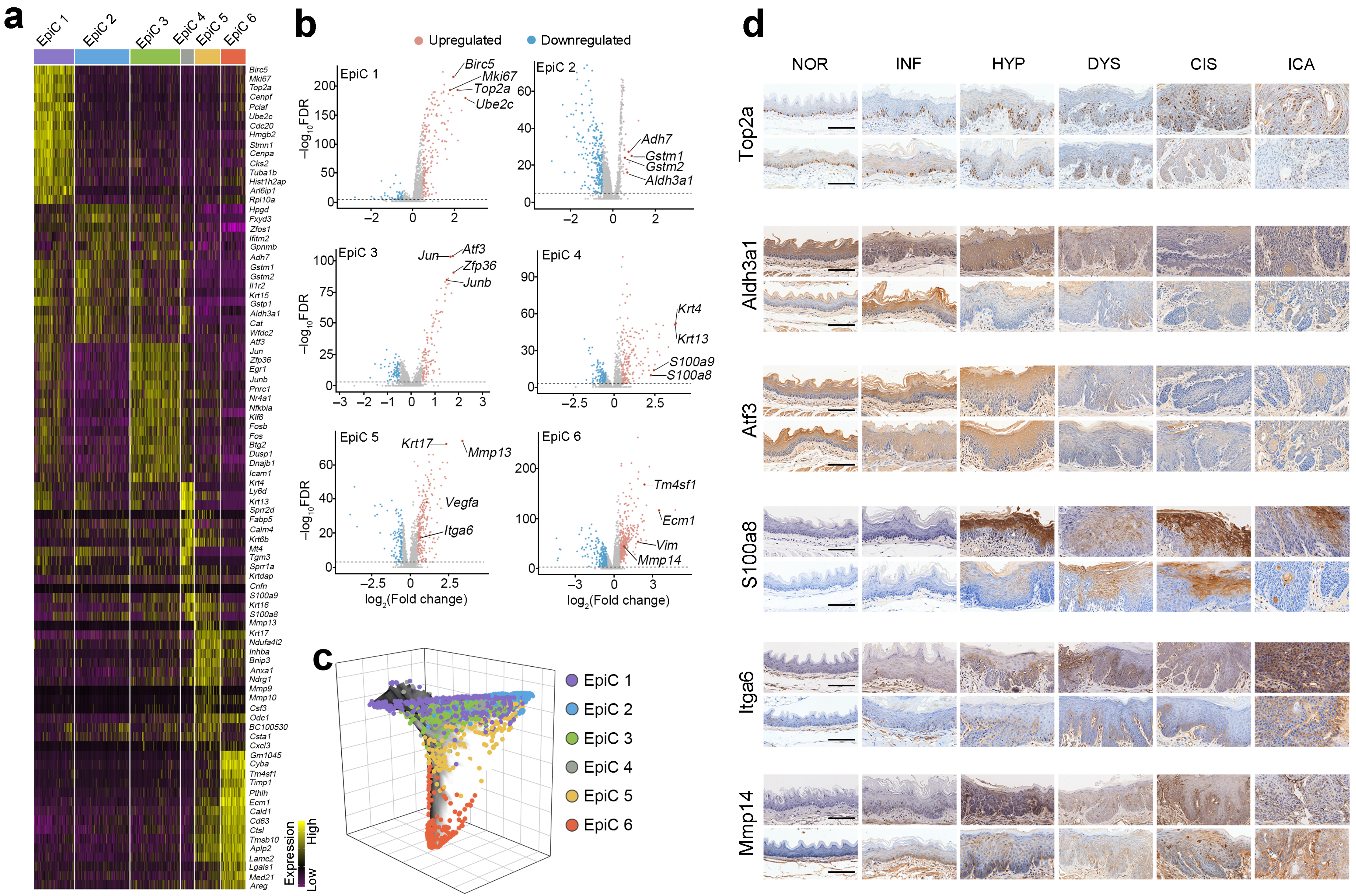
a
EpiC 1
EpiC 2
EpiC 3
EpiC 4
EpiC 5
EpiC 6
Birc5
Mki67
Top2a
Cenpf
Pclaf
Ube2c
Cdc20
Hmgb2
Stmn1
Cenpa
Cks2
Tuba1b
Hist1h2ap
Arl6ip1
Rpl10a
Hpgd
Fxyd3
Zfos1
Ifitm2
Gpnmb
Adh7
Gstm1
Gstm2
Il1r2
Krt15
Gstp1
Aldh3a1
Cat
Wfdc2
Atf3
Jun
Zfp36
Egr1
Junb
Pnrc1
Nr4a1
Nfkbia
Klf6
Fosb
Fos
Btg2
Dusp1
Dnajb1
Icam1
Krt4
Ly6d
Krt13
Sprr2d
Fabp5
Calm4
Krt6b
Mt4
Tgm3
Sprr1a
Krtdap
Cnfn
S100a9
Krt16
S100a8
Mmp13
Krt17
Ndufa4l2
Inhba
Bnip3
Anxa1
Ndrg1
Mmp9
Mmp10
Csf3
Odc1
BC100530
Csta1
Cxcl3
Gm1045
Cyba
Tm4sf1
Timp1
Pthlh
Ecm1
Cald1
Cd63
Ctsl
Tmsb10
Aplp2
Lamc2
Lgals1
Med21
Areg
Expression
High
Low
b
Upregulated
Downregulated
EpiC 1
Birc5
Mki67
Top2a
Ube2c
EpiC 2
Adh7
Gstm1
Gstm2
Aldh3a1
EpiC 3
Jun
Atf3
Zfp36
Junb
EpiC 4
Krt4
Krt13
S100a9
S100a8
EpiC 5
Krt17
Mmp13
Vegfa
Itga6
EpiC 6
Tm4sf1
Ecm1
Vim
Mmp14
−log10FDR
log2(Fold change)
c
EpiC 1
EpiC 2
EpiC 3
EpiC 4
EpiC 5
EpiC 6
d
NOR
INF
HYP
DYS
CIS
ICA
Top2a
Aldh3a1
Atf3
S100a8
Itga6
Mmp14

Supplementary Fig. 2 Expression of marker genes and upregulated genes in epithelial clusters. **a** Heatmap of marker gene expression of 6 epithelial cells clusters. The top 15 marker genes of each cluster are shown in the figure. **b** Volcano plots showing differential expressed genes of each epithelial cluster compared to the rest clusters, respectively. Red dots indicate significantly up-regulated genes (Fold change > 1.40) and blue ones indicate down-regulated genes (Fold change < 0.71). Selected highly expressed genes are labeled. The dashed line indicates FDR = 0.05. **c** Visualization in three-dimension of all epithelial cells using first 3 diffusion components. Each dot represents a cell colored by cluster. **d** IHC staining of protein levels displaying additional visual fields to Fig. 2f, produced by the selective 6 genes in mouse esophageal tissues with different lesions (from Stage NOR to Stage ICA). Scale bars, 100 μm.

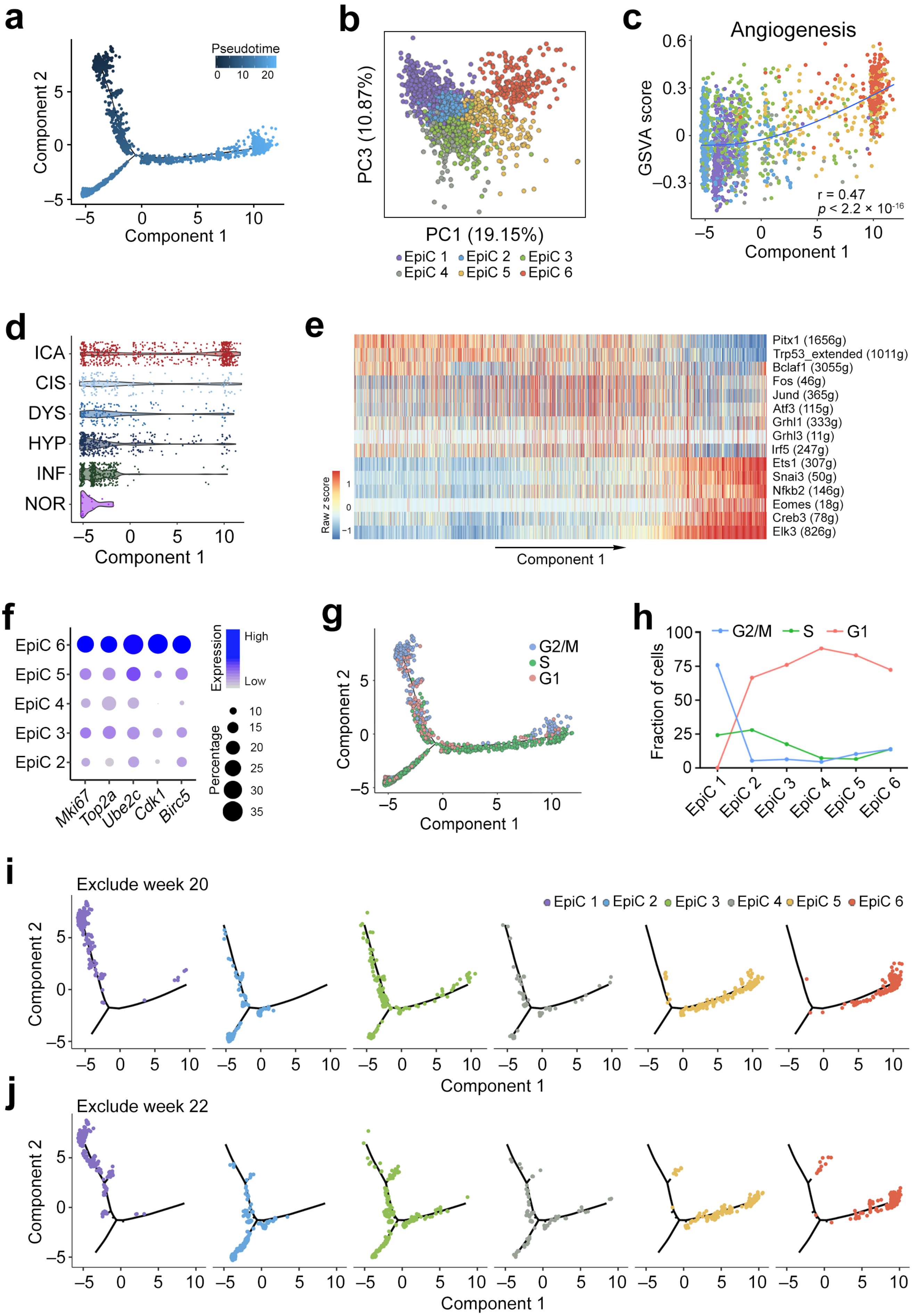

a
Pseudotime
Component 2
Component 1
b
PC3 (10.87%)
PC1 (19.15%)
EpiC 1 EpiC 2 EpiC 3
EpiC 4 EpiC 5 EpiC 6
c
Angiogenesis
GSVA score
r = 0.47
p < 2.2 × 10^-16
Component 1
d
ICA
CIS
DYS
HYP
INF
NOR
Component 1
e
Pitx1 (1656g)
Trp53_extended (1011g)
Bclaf1 (3055g)
Fos (46g)
Jund (365g)
Atf3 (115g)
Grhl1 (333g)
Grhl3 (11g)
Irf5 (247g)
Ets1 (307g)
Snai3 (50g)
Nfkb2 (146g)
Eomes (18g)
Creb3 (78g)
Elk3 (826g)
Raw z score
Component 1
f
EpiC 6
EpiC 5
EpiC 4
EpiC 3
EpiC 2
Mki67 Top2a Ube2c Cdk1 Birc5
Expression
High
Low
Percentage
g
G2/M
S
G1
Component 2
Component 1
h
G2/M S G1
Fraction of cells
EpiC 1 EpiC 2 EpiC 3 EpiC 4 EpiC 5 EpiC 6
i
Exclude week 20
EpiC 1 EpiC 2 EpiC 3 EpiC 4 EpiC 5 EpiC 6
Component 2
Component 1
j
Exclude week 22
Component 2
Component 1

Supplementary Fig. 3 Additional details in epithelial cell states across clusters and pathological stages. **a** Pseudotime trajectory over epithelial cells in a two-dimensional statespace. Each dot represents a cell; the color indicates pseudo time value. **b** PCA plot of epithelial cells colored by clusters as defined in Fig. 2a. Each dot represents a cell. **c** Correlation between Angiogenesis pathway enrichment scores and Component 1 values of single cells. Color indicates clusters designated in Fig. 2a. **d** Violin plots of the distribution of Component 1 values across pathological stages. **e** Heatmap of the area under the curve (AUC) scores of expression regulation by transcription factors, as estimated using SCENIC. Shown are the selected transcription factors having significant difference in expression regulation estimates of epithelial cells ordered by Component 1 value. **f** Bubble plot showing expression levels of the proliferation-related genes across EpiC 2–6. Size of dots represents the percentage of cells expressing the gene; color scale shows the average expression level. **g** Pseudotime trajectory over epithelial cells in a two-dimensional state space. Each dot represents a single cell and each color represents different cell cycle status calculated by Seurat. **h** Line chart displaying changing trend of proportion of cells at G1, S and G2/M status of epithelial clusters, respectively. **i, j** Pseudotime trajectory excluding cells from week 20 (**i**) and week 22 (**j**), displayed by cluster. Each dot represents a cell.

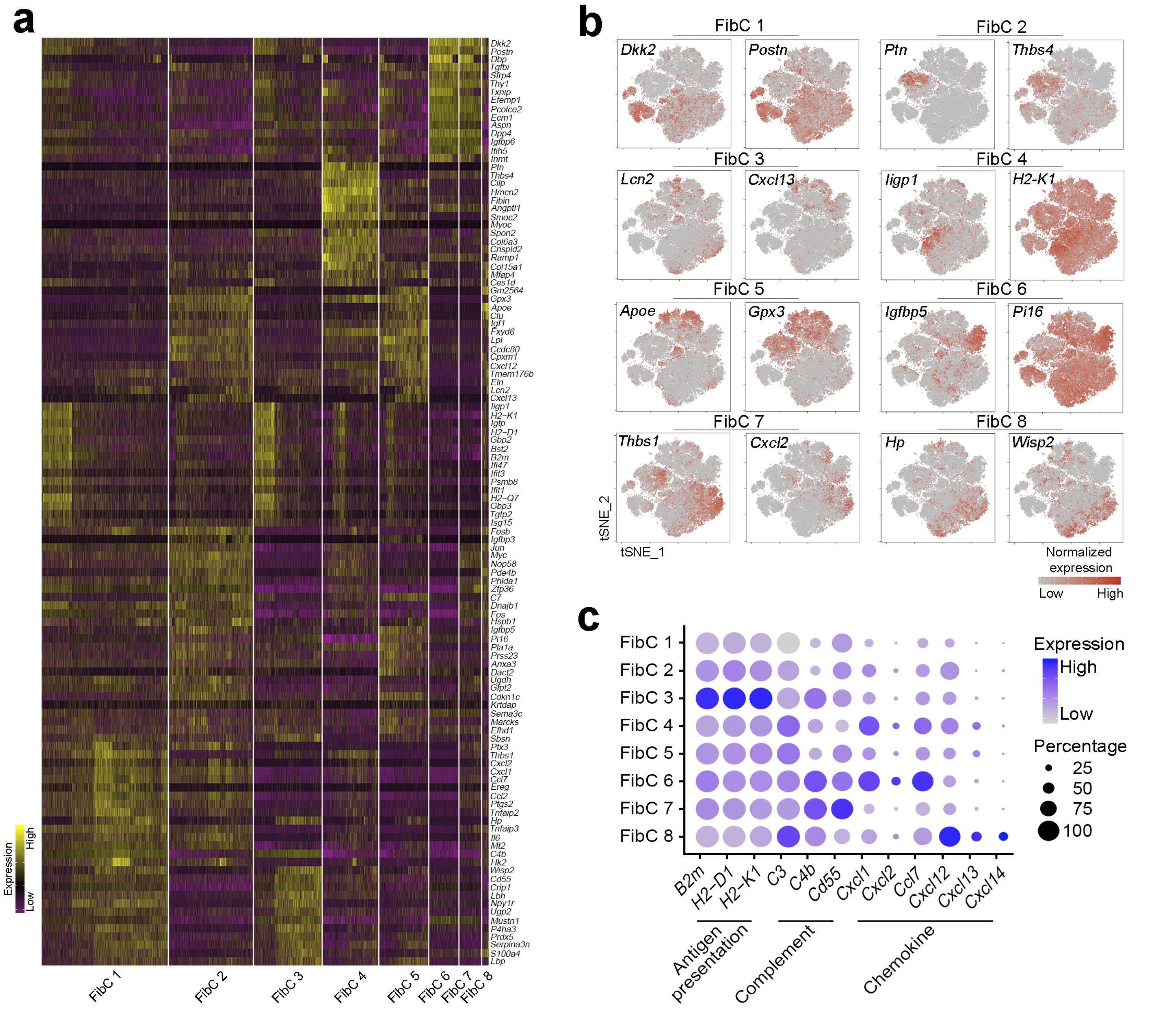

Supplementary Fig. 4 Expression level of marker gene and genes with specific function in fibroblasts. **a** Heatmap showing marker gene expression of 6 fibroblast clusters. Top 15 genes of each cluster are shown. **b** tSNE plots of scale normalized expression of selected marker genes from (**a**) across fibroblast clusters. **c** Bubble plot displaying expression level of genes involved in antigen presentation, complement and chemokine across 8 fibroblast clusters. Size of dots represents percentage of cells expressing corresponding genes in the cluster.

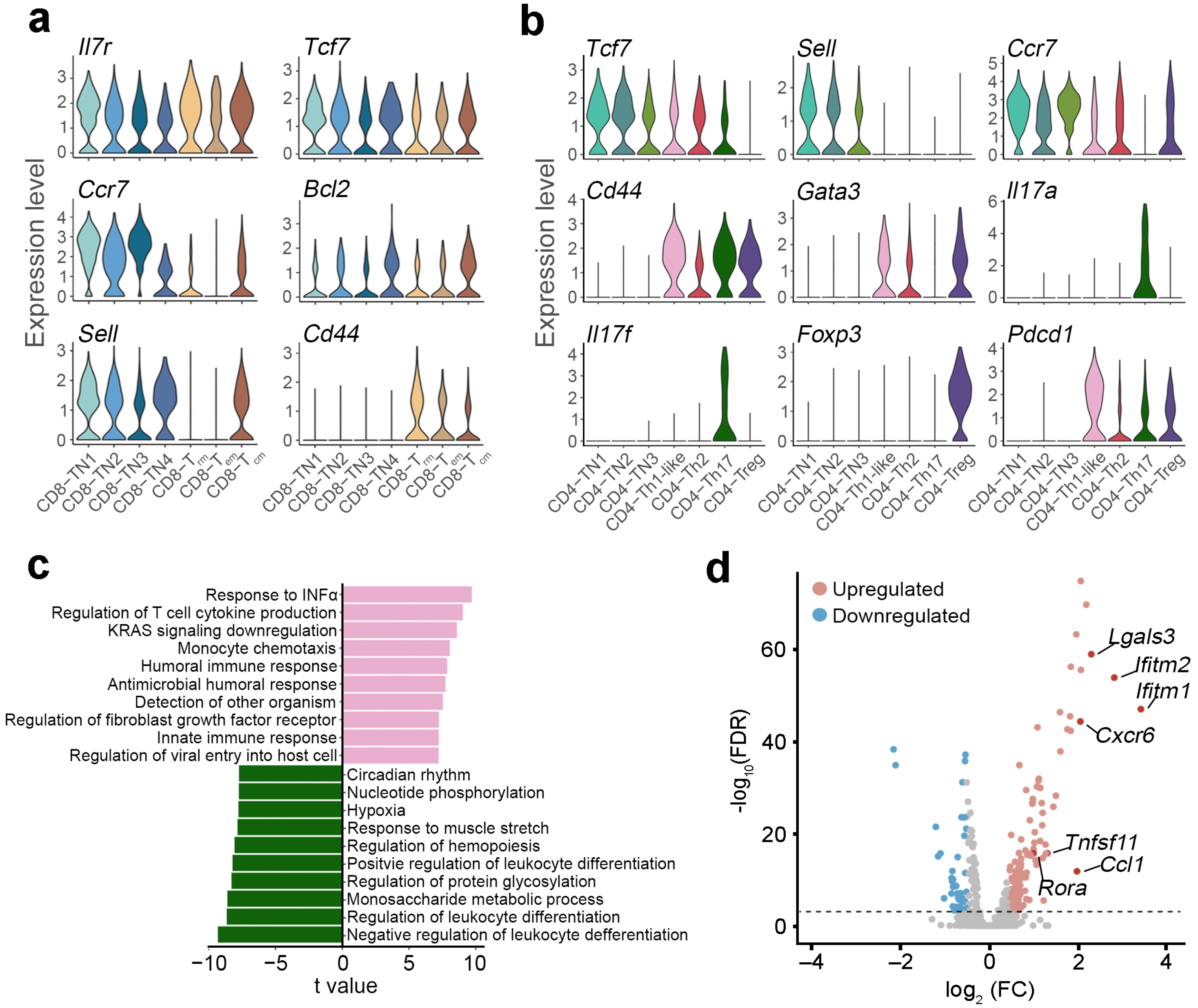

Supplementary Fig. 5 Additional data in expression characters of T cell clusters and clustering of B cells. **a** Violin plots demonstrating marker gene expression of naïve (*Il7r*, *Tcf7* and *Ccr7*) and memory (*Bcl2*, *Sell* and *Cd44*) CD8+ T cell across clusters. **b** Violin plots demonstrating marker gene expression of naïve (*Tcf7*, *Sell* and *Ccr7*), highly differentiated (*Cd44* and *Gata3*), Th17 (*Il17a* and *Il17f*), Treg (*Foxp3*) and exhausted (*Pdcd1*) CD4+ T cell across clusters. **c** Bar plot showing top 10 differently enriched pathways between CD4-Th1 and CD4-Th17 using GSVA. Each bar represents t value of pathway. **d** Volcano plot of up- and down-regulated genes between CD4-Th1 and CD4-Th2. Differential analysis uses the same cut-off points and is in the same format as in Supplementary Fig. 2b.

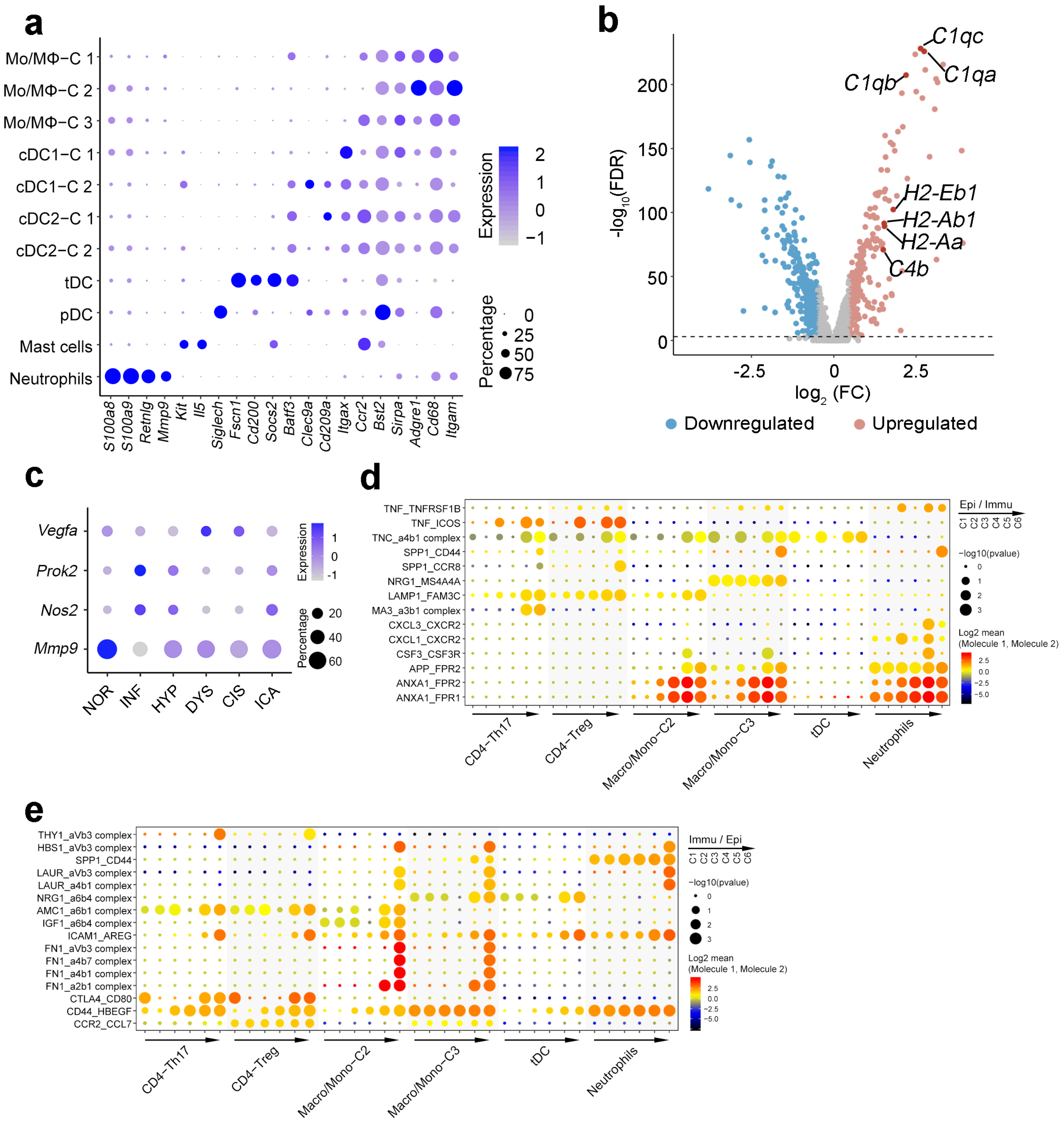

Supplementary Fig. 6 Additional expression data of myeloid cells. **a** Bubble plot showing marker gene expression in myeloid cell clusters. **b** Volcano plot of up- and down-regulated genes between Mo/MΦ-C 1 and Mo/MΦ-C 3. Differential analysis uses the same cut-off points and is in the same format as in Supplementary Fig. 2b. **c** Bubble plot showing selected gene expression in neutrophils along 6 pathological stages. **d,e** Bubble plots depicting selected epithelial to immune (**d**) and immune to epithelial (**e**) interactions. The size of dot indicates p-values and color indicates the means of the receptor/ligand pairs between two clusters.

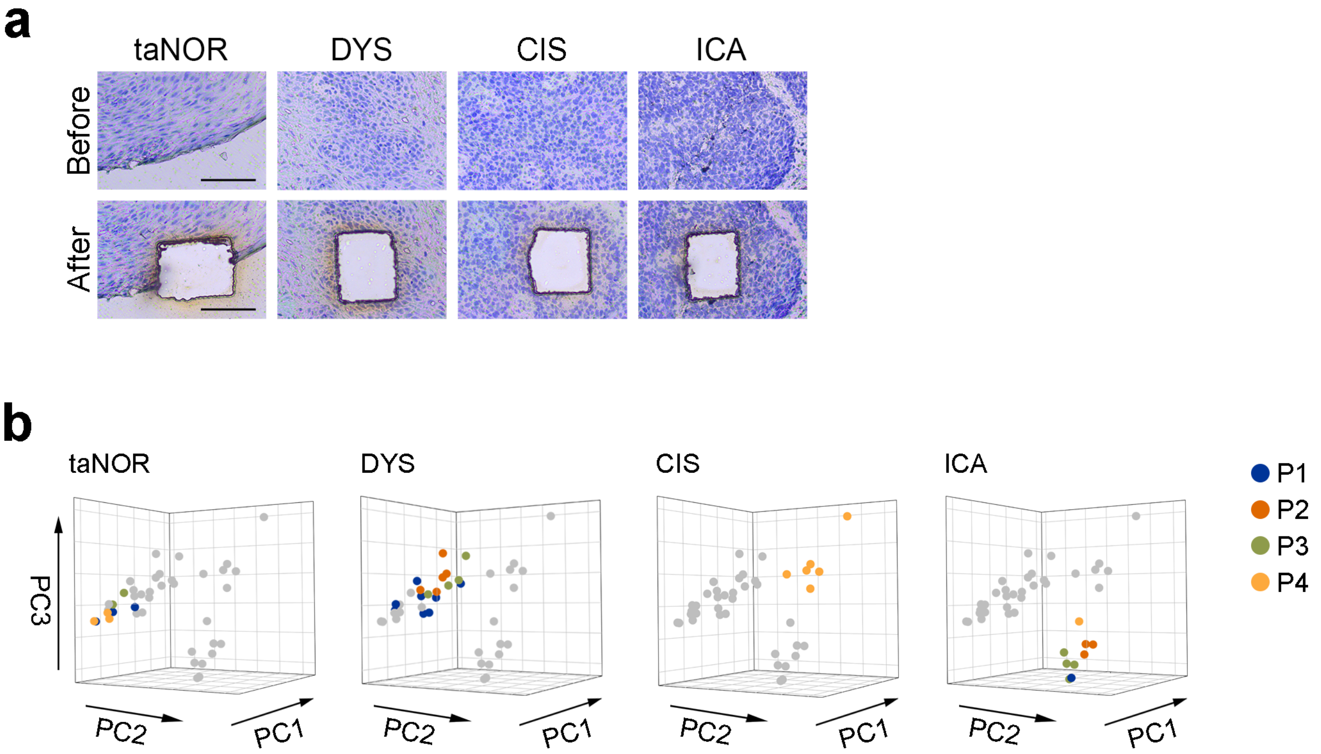

Supplementary Fig. 7 Microdissection images and gene expression pattern in human epithelial lesions. **a** Cresyl violet acetate (0.05%) staining of esophageal epithelia sections of different pathological stages before and after dissection using laser capture microdissection (LCM). Tissues from at least 6 visual fields were captured in each stage. Scale bars, 100 μm. **b** PCA plots showing the transcriptomic difference of the LCM samples divided by pathological stages, colored by individuals.

Supplementary Table 1. Genes differentially expressed in each subcluster of epithelial cells

Cluster	Genes	*P* value	FDR	log2(avg diff)	% cells 1	% cells 2
EpiC 1	*Ccna2*	3.31E-229	4.90E-225	1.457	91.70%	10.40%
EpiC 1	*Birc5*	1.39E-220	2.06E-216	1.964	97.10%	16.30%
EpiC 1	*Cdca3*	6.17E-207	9.15E-203	1.523	92.90%	14.80%
EpiC 1	*Mki67*	7.20E-198	1.07E-193	1.808	92.30%	15.60%
EpiC 1	*Top2a*	1.01E-197	1.50E-193	2.172	92.90%	17.50%
EpiC 1	*Cdk1*	1.74E-195	2.58E-191	1.486	90.00%	13.80%
EpiC 1	*Nusap1*	2.21E-194	3.28E-190	1.073	72.60%	4.80%
EpiC 1	*Pbk*	5.75E-194	8.52E-190	1.001	80.20%	7.80%
EpiC 1	*Cdca8*	1.05E-193	1.56E-189	1.493	96.20%	21.20%
EpiC 1	*Ckap2l*	4.59E-192	6.80E-188	1.390	89.40%	14.60%
EpiC 1	*Cenpf*	9.41E-191	1.39E-186	1.726	83.20%	9.70%
EpiC 1	*Casc5*	6.98E-190	1.03E-185	0.915	76.40%	6.50%
EpiC 1	*2810417H13Rik*	2.38E-188	3.52E-184	1.933	90.90%	15.20%
EpiC 1	*Ube2c*	5.30E-184	7.85E-180	2.549	93.20%	19.60%
EpiC 1	*Aurkb*	5.53E-184	8.19E-180	0.967	74.90%	7.30%
EpiC 1	*Racgap1*	6.44E-184	9.54E-180	1.041	82.90%	10.90%
EpiC 1	*Esco2*	4.70E-182	6.96E-178	0.781	68.10%	4.30%
EpiC 1	*Anln*	8.41E-180	1.25E-175	1.171	81.10%	10.10%
EpiC 1	*Prc1*	1.62E-178	2.40E-174	1.514	86.70%	15.10%
EpiC 1	*Kif11*	3.08E-176	4.56E-172	0.898	69.60%	5.40%
EpiC 1	*Ect2*	5.97E-174	8.84E-170	0.941	78.50%	9.50%
EpiC 1	*Spc24*	1.03E-172	1.53E-168	1.083	83.80%	14.40%
EpiC 1	*Hmmr*	2.70E-172	4.00E-168	1.044	74.90%	7.80%
EpiC 1	*Ccnb1*	9.20E-172	1.36E-167	1.266	79.90%	10.40%
EpiC 1	*Ccnb2*	7.70E-171	1.14E-166	1.212	85.30%	12.70%
EpiC 1	*Spc25*	3.83E-166	5.68E-162	0.707	70.80%	7.00%
EpiC 1	*Cdc20*	5.92E-166	8.78E-162	1.781	8.91E-01	1.93E-01
EpiC 1	*Kif22*	4.38E-165	6.48E-161	0.660	68.70%	6.20%
EpiC 1	*Kifc1*	1.50E-164	2.23E-160	0.671	67.60%	5.80%
EpiC 1	*Tacc3*	1.08E-163	1.61E-159	0.992	83.50%	14.60%
EpiC 1	*Plk1*	2.57E-163	3.81E-159	0.830	67.80%	6.00%
EpiC 1	*Hmgb2*	2.20E-162	3.26E-158	2.363	99.70%	79.20%
EpiC 1	*Stmn1*	1.04E-158	1.54E-154	1.858	97.10%	42.80%
EpiC 1	*Tpx2*	3.03E-156	4.49E-152	1.156	85.50%	17.60%
EpiC 1	*Kif23*	8.79E-156	1.30E-151	0.910	72.90%	9.00%
EpiC 1	*Kif20b*	1.84E-155	2.72E-151	0.737	71.40%	8.00%
EpiC 1	*Kif15*	5.59E-155	8.28E-151	0.574	64.30%	5.30%
EpiC 1	*Incenp*	2.70E-154	4.00E-150	1.128	85.80%	19.00%

Cluster	Genes	*P* value	FDR	log2(avg diff)	% cells 1	% cells 2
EpiC 1	*Smc2*	4.48E-154	6.63E-150	1.377	92.90%	28.90%
EpiC 1	*Ncapd2*	1.21E-152	1.79E-148	0.730	77.60%	11.60%
EpiC 1	*Cenpe*	2.11E-152	3.12E-148	1.176	75.20%	10.70%
EpiC 1	*Shcbp1*	1.63E-149	2.42E-145	0.593	67.60%	7.10%
EpiC 1	*Melk*	1.15E-148	1.71E-144	0.592	66.10%	6.90%
EpiC 1	*Cenpa*	2.39E-144	3.55E-140	1.704	86.10%	20.40%
EpiC 1	*Tk1*	2.05E-141	3.04E-137	1.446	88.20%	25.60%
EpiC 1	*Depdc1a*	6.50E-141	9.62E-137	0.532	55.20%	3.50%
EpiC 1	*Smc4*	2.72E-140	4.03E-136	1.531	94.10%	43.00%
EpiC 1	*Hmgb1*	3.45E-140	5.11E-136	1.394	100.00%	98.50%
EpiC 1	*H2afz*	1.37E-138	2.03E-134	1.552	100.00%	97.90%
EpiC 1	*Kif20a*	2.59E-137	3.84E-133	0.594	63.10%	6.70%
EpiC 1	*Aspm*	4.79E-137	7.09E-133	0.779	55.80%	4.00%
EpiC 1	*Bub1b*	7.04E-136	1.04E-131	0.579	62.80%	6.60%
EpiC 1	*Asf1b*	1.04E-134	1.55E-130	0.699	70.80%	10.90%
EpiC 1	*Rad51*	3.32E-134	4.92E-130	0.651	69.60%	10.10%
EpiC 1	*Nuf2*	1.06E-131	1.57E-127	0.488	58.40%	5.40%
EpiC 1	*Cenpm*	3.35E-130	4.96E-126	0.666	69.30%	11.20%
EpiC 1	*Hmgn2*	5.59E-130	8.28E-126	1.658	98.80%	67.20%
EpiC 1	*Cit*	1.11E-129	1.64E-125	0.590	65.50%	8.20%
EpiC 1	*Cks2*	9.00E-129	1.33E-124	1.652	98.50%	66.30%
EpiC 1	*Hist1h1b*	8.95E-128	1.33E-123	1.087	65.20%	8.80%
EpiC 1	*Lockd*	1.01E-127	1.49E-123	0.807	72.00%	12.40%
EpiC 1	*Ncapg*	2.44E-126	3.61E-122	0.512	61.90%	7.30%
EpiC 1	*Ckap2*	3.36E-125	4.97E-121	0.789	71.40%	13.30%
EpiC 1	*Aurka*	4.22E-125	6.25E-121	0.662	63.70%	8.80%
EpiC 1	*Cdca2*	9.20E-125	1.36E-120	0.492	55.20%	5.10%
EpiC 1	*Ccdc34*	1.58E-124	2.34E-120	1.129	93.20%	39.40%
EpiC 1	*Tuba1b*	5.11E-121	7.58E-117	1.884	99.10%	85.50%
EpiC 1	*Cep55*	9.26E-119	1.37E-114	0.606	61.40%	7.80%
EpiC 1	*Kif2c*	4.65E-118	6.88E-114	0.545	54.90%	5.70%
EpiC 1	*Ptma*	5.42E-118	8.02E-114	1.057	100.00%	99.70%
EpiC 1	*Diaph3*	1.60E-117	2.37E-113	0.583	61.90%	8.70%
EpiC 1	*Psrc1*	2.02E-117	2.99E-113	0.592	54.60%	5.60%
EpiC 1	*Mad2l1*	1.58E-114	2.34E-110	0.596	72.00%	15.00%
EpiC 1	*Gm10282*	2.28E-112	3.37E-108	1.252	91.20%	40.80%
EpiC 1	*Sgol2a*	2.49E-111	3.69E-107	0.540	63.10%	9.70%
EpiC 1	*Hist1h2ap*	2.64E-110	3.90E-106	2.188	77.00%	22.60%
EpiC 1	*Cenpw*	3.03E-110	4.49E-106	0.800	83.80%	24.50%
EpiC 1	*H2afv*	4.39E-110	6.50E-106	1.435	96.80%	83.70%
EpiC 1	*Rad51ap1*	5.71E-110	8.47E-106	0.499	56.60%	7.10%

Cluster	Genes	*P* value	FDR	log2(avg diff)	% cells 1	% cells 2
EpiC 1	*Knstrn*	1.09E-109	1.61E-105	0.758	76.10%	18.00%
EpiC 1	*Tubb5*	3.12E-109	4.62E-105	1.549	99.70%	91.60%
EpiC 1	*Cdkn3*	9.85E-109	1.46E-104	0.704	60.20%	9.00%
EpiC 1	*H2afx*	2.12E-108	3.14E-104	1.420	90.00%	50.50%
EpiC 1	*Tubb4b*	2.72E-108	4.03E-104	1.623	99.10%	86.40%
EpiC 1	*Cks1b*	2.84E-105	4.20E-101	0.979	95.90%	54.50%
EpiC 1	*Clspn*	6.07E-102	8.99E-98	0.528	58.70%	9.10%
EpiC 1	*Rrm1*	8.69E-99	1.29E-94	0.860	88.20%	34.90%
EpiC 1	*Hist1h2ae*	1.11E-95	1.64E-91	1.580	72.00%	22.00%
EpiC 1	*Hist1h2ao*	1.21E-94	1.80E-90	0.618	45.70%	4.90%
EpiC 1	*Dek*	2.96E-94	4.38E-90	1.111	96.80%	75.20%
EpiC 1	*Lmnb1*	8.92E-93	1.32E-88	0.964	89.40%	43.30%
EpiC 1	*Nrm*	4.78E-92	7.08E-88	0.781	75.80%	24.80%
EpiC 1	*C330027C09Rik*	4.78E-91	7.08E-87	0.532	57.80%	10.40%
EpiC 1	*Dlgap5*	2.20E-90	3.26E-86	0.505	54.30%	9.00%
EpiC 1	*Ube2s*	8.10E-90	1.20E-85	1.186	97.10%	74.00%
EpiC 1	*Tyms*	3.49E-89	5.17E-85	0.890	79.10%	28.70%
EpiC 1	*Tmpo*	1.16E-83	1.72E-79	0.942	94.70%	66.10%
EpiC 1	*Cdkn2d*	2.22E-83	3.28E-79	0.666	72.90%	22.90%
EpiC 1	*Ptn*	8.48E-83	1.26E-78	1.216	88.50%	42.90%
EpiC 1	*2700094K13Rik*	2.51E-81	3.72E-77	0.877	95.90%	77.50%
EpiC 1	*Hn1*	7.41E-81	1.10E-76	0.962	96.20%	72.70%
EpiC 1	*Uhrf1*	1.02E-80	1.51E-76	0.539	60.20%	13.10%
EpiC 1	*Cdc25b*	6.89E-79	1.02E-74	0.525	61.40%	14.80%
EpiC 1	*Dbf4*	4.13E-78	6.12E-74	0.593	74.00%	25.10%
EpiC 1	*Rfc5*	4.63E-78	6.86E-74	0.532	76.10%	26.30%
EpiC 1	*Arl6ip1*	1.12E-77	1.67E-73	1.653	96.80%	83.30%
EpiC 1	*Fam83d*	2.57E-76	3.81E-72	0.556	59.30%	13.80%
EpiC 1	*Mis18a*	7.48E-76	1.11E-71	0.531	72.90%	25.70%
EpiC 1	*Nucks1*	3.37E-74	5.00E-70	0.918	95.60%	69.10%
EpiC 1	*Trim59*	2.08E-73	3.09E-69	0.704	79.10%	30.60%
EpiC 1	*Rrm2*	3.61E-71	5.35E-67	0.883	66.70%	21.30%
EpiC 1	*Kpna2*	5.84E-71	8.65E-67	1.179	84.40%	47.80%
EpiC 1	*Fen1*	6.54E-70	9.69E-66	0.616	71.70%	26.50%
EpiC 1	*Pmf1*	9.76E-70	1.45E-65	0.531	73.20%	27.30%
EpiC 1	*Rad21*	1.37E-65	2.03E-61	0.887	91.40%	59.00%
EpiC 1	*Dnph1*	9.65E-64	1.43E-59	0.538	78.20%	33.90%
EpiC 1	*Ezh2*	1.47E-63	2.17E-59	0.585	75.80%	32.30%
EpiC 1	*Atad2*	3.55E-63	5.26E-59	0.559	63.70%	20.10%
EpiC 1	*Calm2*	3.69E-63	5.47E-59	0.773	99.40%	93.60%
EpiC 1	*Hist1h1e*	1.30E-61	1.92E-57	0.730	63.40%	20.80%

Cluster	Genes	*P* value	FDR	log2(avg diff)	% cells 1	% cells 2
EpiC 1	*Lig1*	4.99E-61	7.39E-57	0.595	61.10%	18.90%
EpiC 1	*Anp32e*	7.18E-61	1.06E-56	0.726	90.30%	61.70%
EpiC 1	*Tcf19*	1.74E-58	2.57E-54	0.533	46.90%	11.20%
EpiC 1	*Lsm2*	4.58E-58	6.79E-54	0.683	91.20%	69.20%
EpiC 1	*Lsm3*	8.11E-58	1.20E-53	0.623	90.00%	64.10%
EpiC 1	*Mcm5*	1.17E-56	1.73E-52	0.537	64.60%	22.50%
EpiC 1	*Lbr*	2.06E-56	3.06E-52	0.534	74.90%	31.10%
EpiC 1	*Ckap5*	3.63E-55	5.38E-51	0.626	72.00%	31.60%
EpiC 1	*Dut*	4.07E-55	6.03E-51	0.855	89.40%	59.20%
EpiC 1	*Rangap1*	4.85E-55	7.19E-51	0.617	86.70%	53.20%
EpiC 1	*Bub3*	6.28E-55	9.31E-51	0.701	90.90%	70.80%
EpiC 1	*Ran*	4.21E-54	6.24E-50	0.554	100.00%	98.20%
EpiC 1	*Hnrnpa3*	8.98E-54	1.33E-49	0.565	100.00%	99.80%
EpiC 1	*Hist1h2ab*	2.97E-52	4.40E-48	0.550	41.60%	9.10%
EpiC 1	*Gpsm2*	1.10E-50	1.62E-46	0.575	77.00%	37.80%
EpiC 1	*Nde1*	3.42E-50	5.07E-46	0.506	74.30%	36.80%
EpiC 1	*Lsm5*	1.11E-49	1.65E-45	0.566	92.30%	71.60%
EpiC 1	*Usp1*	1.23E-49	1.82E-45	0.523	85.30%	46.50%
EpiC 1	*Anp32b*	4.47E-49	6.63E-45	0.583	98.20%	95.10%
EpiC 1	*Dnajc9*	7.09E-47	1.05E-42	0.494	73.70%	36.70%
EpiC 1	*Banf1*	7.84E-45	1.16E-40	0.490	98.80%	93.60%
EpiC 1	*Sae1*	4.98E-44	7.38E-40	0.534	94.40%	74.70%
EpiC 1	*Hmgb3*	1.36E-42	2.02E-38	0.503	81.40%	48.90%
EpiC 1	*Psip1*	1.68E-42	2.49E-38	0.554	84.40%	56.50%
EpiC 1	*Dnmt1*	2.60E-42	3.85E-38	0.567	70.50%	35.10%
EpiC 1	*Smc1a*	5.83E-41	8.63E-37	0.549	90.00%	68.80%
EpiC 1	*Ddx39b*	3.14E-40	4.65E-36	0.530	96.80%	85.40%
EpiC 1	*Tipin*	3.44E-40	5.10E-36	0.560	74.00%	42.30%
EpiC 1	*Mcm7*	7.22E-40	1.07E-35	0.492	75.50%	39.30%
EpiC 1	*Hells*	1.93E-39	2.86E-35	0.537	63.10%	27.30%
EpiC 1	*Rnaseh2c*	4.85E-39	7.19E-35	0.508	90.90%	70.20%
EpiC 1	*Ywhah*	8.60E-38	1.27E-33	0.524	97.90%	90.80%
EpiC 1	*Ssrp1*	1.24E-37	1.83E-33	0.510	92.30%	81.40%
EpiC 1	*Tuba1c*	6.99E-37	1.04E-32	0.672	96.80%	87.40%
EpiC 1	*Dctpp1*	1.29E-36	1.91E-32	0.598	87.00%	67.90%
EpiC 1	*Tap1*	4.74E-36	7.02E-32	0.568	71.70%	37.10%
EpiC 1	*Ubald2*	5.05E-36	7.48E-32	0.535	93.50%	81.60%
EpiC 1	*Psmb9*	9.14E-36	1.35E-31	0.636	67.30%	34.60%
EpiC 1	*Rbbp7*	3.53E-35	5.23E-31	0.485	92.00%	78.00%
EpiC 1	*Mt2*	4.25E-35	6.30E-31	0.905	99.40%	91.80%
EpiC 1	*Gmnn*	4.46E-34	6.60E-30	0.525	69.90%	40.90%

Cluster	Genes	*P* value	FDR	log2(avg diff)	% cells 1	% cells 2
EpiC 1	*Smc6*	2.36E-33	3.50E-29	0.490	88.50%	66.20%
EpiC 1	*H3f3b*	7.43E-33	1.10E-28	0.508	100.00%	99.90%
EpiC 1	*Hp1bp3*	1.33E-31	1.98E-27	0.557	92.30%	72.50%
EpiC 1	*Ptms*	1.15E-29	1.70E-25	0.598	97.60%	95.60%
EpiC 1	*Krt15*	3.16E-29	4.68E-25	0.981	85.80%	59.10%
EpiC 1	*H2-Q4*	5.64E-29	8.35E-25	0.621	76.10%	48.80%
EpiC 1	*Igfbp2*	2.01E-27	2.99E-23	0.704	78.80%	50.90%
EpiC 1	*H1f0*	4.80E-27	7.11E-23	0.747	87.30%	67.50%
EpiC 1	*Ephx1*	6.76E-27	1.00E-22	0.544	74.30%	48.30%
EpiC 1	*Them5*	3.28E-23	4.86E-19	0.725	76.70%	49.80%
EpiC 1	*Gclc*	3.72E-22	5.52E-18	0.704	60.80%	34.40%
EpiC 1	*Pcna*	6.32E-22	9.36E-18	0.568	76.70%	58.90%
EpiC 1	*Hsp90aa1*	9.46E-22	1.40E-17	0.535	98.50%	98.00%
EpiC 1	*Slbp*	4.30E-20	6.37E-16	0.505	72.90%	54.60%
EpiC 1	*H2-Q7*	6.28E-20	9.31E-16	0.623	67.60%	45.40%
EpiC 1	*Ifi27l2a*	1.48E-19	2.19E-15	1.126	66.70%	42.60%
EpiC 1	*Ptgr1*	1.54E-17	2.29E-13	0.642	80.20%	58.30%
EpiC 1	*Gstm1*	2.15E-17	3.19E-13	0.600	89.70%	74.70%
EpiC 1	*Cxcl10*	2.37E-17	3.51E-13	0.799	35.10%	14.70%
EpiC 1	*Aldh3a1*	3.39E-17	5.02E-13	0.925	73.70%	50.80%
EpiC 1	*Apoe*	4.13E-16	6.12E-12	0.500	57.20%	35.10%
EpiC 1	*Nqo1*	1.43E-14	2.11E-10	0.756	79.10%	64.60%
EpiC 1	*Adh7*	2.73E-14	4.04E-10	0.539	80.20%	58.10%
EpiC 1	*Cbr3*	1.16E-13	1.72E-09	0.624	78.20%	62.50%
EpiC 1	*Isg15*	6.87E-11	1.02E-06	0.493	41.60%	24.90%
EpiC 2	*Rps18*	2.36E-65	3.49E-61	0.516	100.00%	99.70%
EpiC 2	*Rpl10a*	1.28E-63	1.89E-59	0.521	100.00%	99.90%
EpiC 2	*Rpl3*	6.74E-56	9.98E-52	0.489	100.00%	99.70%
EpiC 2	*Hpgd*	2.39E-47	3.54E-43	1.215	75.40%	40.10%
EpiC 2	*Eif3f*	8.08E-47	1.20E-42	0.514	99.60%	97.40%
EpiC 2	*Fxyd3*	6.58E-40	9.75E-36	0.609	98.90%	93.70%
EpiC 2	*Zfos1*	6.41E-34	9.50E-30	0.534	89.00%	77.30%
EpiC 2	*Ifitm2*	7.57E-32	1.12E-27	0.561	98.90%	97.50%
EpiC 2	*Gpnmb*	2.37E-31	3.51E-27	0.546	78.20%	49.60%
EpiC 2	*Adh7*	1.98E-30	2.93E-26	0.750	82.70%	55.10%
EpiC 2	*Gstm1*	3.20E-28	4.74E-24	0.894	89.40%	73.30%
EpiC 2	*Gstm2*	3.61E-27	5.35E-23	0.570	80.30%	56.80%
EpiC 2	*Il1r2*	1.35E-24	2.00E-20	0.785	59.60%	34.20%
EpiC 2	*Rpl22l1*	1.33E-23	1.97E-19	0.493	99.10%	95.40%
EpiC 2	*Krt15*	5.42E-21	8.03E-17	0.660	85.70%	56.60%
EpiC 2	*Gstp1*	7.99E-21	1.18E-16	0.630	99.40%	98.10%

Cluster	Genes	*P* value	FDR	log2(avg diff)	% cells 1	% cells 2
EpiC 2	*Aldh3a1*	3.59E-19	5.33E-15	0.689	72.40%	49.10%
EpiC 2	*Them5*	1.02E-18	1.51E-14	0.515	73.70%	48.30%
EpiC 3	*Atf3*	1.02E-108	1.51E-104	1.765	95.50%	52.10%
EpiC 3	*Jun*	3.00E-108	4.44E-104	1.665	99.50%	85.80%
EpiC 3	*Zfp36*	1.68E-95	2.48E-91	1.794	96.40%	74.50%
EpiC 3	*Egr1*	1.02E-90	1.51E-86	1.580	93.60%	60.20%
EpiC 3	*Junb*	1.10E-89	1.64E-85	1.509	97.40%	92.10%
EpiC 3	*Pnrc1*	4.63E-88	6.86E-84	1.439	96.90%	88.00%
EpiC 3	*Nr4a1*	1.50E-86	2.22E-82	1.601	81.20%	34.40%
EpiC 3	*Nfkbia*	4.54E-86	6.72E-82	1.531	97.60%	88.80%
EpiC 3	*Klf6*	2.80E-83	4.15E-79	1.482	96.20%	74.10%
EpiC 3	*Fosb*	7.88E-83	1.17E-78	1.272	81.90%	35.00%
EpiC 3	*Fos*	5.29E-79	7.83E-75	1.710	95.50%	69.80%
EpiC 3	*Btg2*	3.62E-78	5.37E-74	1.431	94.10%	72.50%
EpiC 3	*Ppp1r15a*	1.93E-73	2.86E-69	1.097	85.50%	49.70%
EpiC 3	*Dusp1*	7.42E-73	1.10E-68	1.432	96.90%	72.30%
EpiC 3	*Tnfaip3*	7.01E-65	1.04E-60	1.074	62.90%	23.00%
EpiC 3	*Ier2*	3.22E-64	4.77E-60	1.258	93.80%	83.70%
EpiC 3	*Dnajb1*	6.06E-64	8.97E-60	1.329	96.00%	88.50%
EpiC 3	*Ifrd1*	1.33E-63	1.96E-59	1.223	91.90%	74.80%
EpiC 3	*Adrb2*	1.19E-62	1.77E-58	1.142	86.50%	55.40%
EpiC 3	*Tsc22d1*	2.12E-62	3.14E-58	1.065	95.00%	84.50%
EpiC 3	*Btg1*	1.69E-61	2.51E-57	0.863	97.60%	86.30%
EpiC 3	*Jund*	2.69E-59	3.98E-55	0.947	97.60%	86.90%
EpiC 3	*Zfand5*	2.46E-58	3.64E-54	1.031	91.20%	76.60%
EpiC 3	*Fosl1*	1.24E-56	1.84E-52	1.185	77.90%	42.30%
EpiC 3	*Nfkbiz*	8.79E-56	1.30E-51	0.978	89.80%	66.70%
EpiC 3	*Ubc*	1.14E-54	1.69E-50	0.931	97.40%	93.10%
EpiC 3	*Bhlhe40*	1.14E-52	1.70E-48	0.935	85.70%	55.00%
EpiC 3	*Rnd3*	2.74E-52	4.06E-48	0.898	79.80%	49.70%
EpiC 3	*Icam1*	1.54E-51	2.28E-47	1.260	78.10%	52.40%
EpiC 3	*Csrnp1*	2.40E-49	3.56E-45	0.573	60.60%	24.60%
EpiC 3	*Adamts1*	2.41E-48	3.57E-44	1.129	66.70%	33.90%
EpiC 3	*Fosl2*	1.57E-47	2.33E-43	0.884	83.10%	62.70%
EpiC 3	*Slc25a25*	3.01E-47	4.46E-43	0.672	60.30%	27.30%
EpiC 3	*Ccnl1*	1.39E-43	2.05E-39	0.681	88.60%	72.70%
EpiC 3	*Klf9*	1.60E-43	2.37E-39	0.711	76.70%	49.70%
EpiC 3	*Klf4*	3.93E-43	5.82E-39	0.845	86.00%	62.20%
EpiC 3	*Plk2*	2.42E-42	3.58E-38	0.868	80.00%	53.30%
EpiC 3	*Trib1*	7.93E-42	1.18E-37	0.683	68.40%	36.30%
EpiC 3	*Ptp4a1*	3.47E-41	5.14E-37	0.692	88.60%	75.40%

Cluster	Genes	*P* value	FDR	log2(avg diff)	% cells 1	% cells 2
EpiC 3	*Klf5*	4.16E-39	6.17E-35	0.698	97.40%	93.50%
EpiC 3	*Ier3*	2.93E-38	4.34E-34	0.885	94.50%	80.80%
EpiC 3	*Zfp36l1*	3.29E-37	4.87E-33	0.988	95.70%	90.90%
EpiC 3	*Stat3*	1.65E-34	2.45E-30	0.601	83.10%	62.60%
EpiC 3	*Pim3*	8.40E-34	1.24E-29	0.609	74.10%	52.20%
EpiC 3	*Zc3h12a*	2.70E-33	4.00E-29	0.604	74.80%	50.80%
EpiC 3	*Tgif1*	7.97E-33	1.18E-28	0.774	77.40%	62.80%
EpiC 3	*Nfil3*	2.34E-32	3.46E-28	0.548	65.10%	40.40%
EpiC 3	*Irf1*	3.80E-32	5.64E-28	0.722	61.30%	36.00%
EpiC 3	*Id3*	9.92E-32	1.47E-27	1.087	82.20%	64.90%
EpiC 3	*Bcl10*	1.07E-31	1.58E-27	0.551	86.00%	73.20%
EpiC 3	*Sqstm1*	1.72E-31	2.55E-27	0.629	91.90%	85.60%
EpiC 3	*Fam110c*	3.64E-30	5.40E-26	0.641	61.80%	36.90%
EpiC 3	*Ets2*	8.30E-30	1.23E-25	0.579	82.90%	63.00%
EpiC 3	*Dnajb9*	1.48E-29	2.20E-25	0.548	65.60%	43.90%
EpiC 3	*Arl4a*	2.52E-29	3.73E-25	0.732	72.90%	56.10%
EpiC 3	*Maff*	3.90E-28	5.78E-24	0.492	53.90%	29.00%
EpiC 3	*Hcar2*	2.16E-27	3.19E-23	0.504	82.20%	66.90%
EpiC 3	*Tob1*	4.75E-27	7.03E-23	0.596	78.40%	60.90%
EpiC 3	*Arc*	1.97E-25	2.92E-21	0.944	46.10%	23.10%
EpiC 3	*4930523C07Rik*	2.47E-25	3.66E-21	0.576	66.30%	44.90%
EpiC 3	*Ehf*	2.49E-25	3.69E-21	0.641	91.20%	70.50%
EpiC 3	*Hspa1a*	3.05E-25	4.52E-21	0.892	88.40%	74.80%
EpiC 3	*Pax9*	6.58E-25	9.74E-21	0.540	88.40%	79.40%
EpiC 3	*Sgk1*	1.46E-24	2.17E-20	0.861	82.70%	70.80%
EpiC 3	*Errfi1*	3.35E-24	4.96E-20	0.796	62.70%	40.80%
EpiC 3	*Socs3*	4.13E-24	6.12E-20	1.021	71.50%	54.30%
EpiC 3	*Cdc42ep3*	8.21E-24	1.22E-19	0.712	66.50%	48.80%
EpiC 3	*Hspb8*	1.94E-23	2.88E-19	0.625	86.00%	77.30%
EpiC 3	*Sdc4*	1.90E-22	2.81E-18	0.840	90.00%	83.10%
EpiC 3	*Sat1*	9.89E-22	1.47E-17	0.828	95.20%	93.40%
EpiC 3	*Chka*	3.62E-21	5.36E-17	0.651	60.30%	38.80%
EpiC 3	*Ifitm3*	5.67E-21	8.40E-17	0.490	99.00%	99.00%
EpiC 3	*Slc6a6*	1.84E-20	2.73E-16	0.657	80.50%	66.10%
EpiC 3	*Hspa1b*	1.09E-19	1.61E-15	0.731	80.30%	68.70%
EpiC 3	*Myc*	2.96E-19	4.38E-15	0.640	78.60%	63.20%
EpiC 3	*Creb5*	3.24E-19	4.80E-15	0.574	44.70%	25.00%
EpiC 3	*Egr4*	6.28E-19	9.30E-15	0.547	18.10%	4.90%
EpiC 3	*Tiparp*	1.04E-18	1.54E-14	0.542	67.90%	52.50%
EpiC 3	*Ccl2*	2.84E-18	4.21E-14	1.191	50.80%	32.50%
EpiC 3	*Phlda1*	4.60E-18	6.82E-14	0.666	86.00%	69.10%

Cluster	Genes	*P* value	FDR	log2(avg diff)	% cells 1	% cells 2
EpiC 3	*Fgfr2*	3.07E-17	4.55E-13	0.495	74.80%	58.70%
EpiC 3	*Cxcl10*	4.23E-17	6.27E-13	0.915	31.80%	14.50%
EpiC 3	*Neat1*	9.98E-17	1.48E-12	0.975	91.90%	85.50%
EpiC 3	*Slc38a2*	1.33E-16	1.97E-12	0.579	96.00%	89.20%
EpiC 3	*Hsph1*	2.31E-16	3.42E-12	0.720	77.40%	65.50%
EpiC 3	*Rassf10*	7.55E-16	1.12E-11	0.538	54.20%	36.60%
EpiC 3	*Pmaip1*	2.03E-15	3.01E-11	0.538	48.20%	31.70%
EpiC 3	*Mt1*	3.35E-15	4.96E-11	0.534	99.00%	98.30%
EpiC 3	*Cyr61*	3.77E-15	5.59E-11	0.957	68.20%	53.20%
EpiC 3	*Gpnmb*	4.63E-15	6.85E-11	0.668	70.10%	53.00%
EpiC 3	*Pde4b*	7.23E-15	1.07E-10	0.566	50.60%	34.50%
EpiC 3	*Clic4*	1.02E-14	1.51E-10	0.487	79.30%	66.20%
EpiC 3	*2810474O19Rik*	1.31E-14	1.94E-10	0.517	73.90%	61.60%
EpiC 3	*Cxcl1*	2.24E-14	3.32E-10	1.213	50.40%	34.40%
EpiC 3	*Adm*	2.62E-14	3.88E-10	0.738	57.00%	39.30%
EpiC 3	*Cxadr*	7.28E-14	1.08E-09	0.641	77.20%	71.20%
EpiC 3	*Dnaja1*	8.86E-14	1.31E-09	0.532	95.00%	93.00%
EpiC 3	*Hspb1*	1.05E-12	1.56E-08	0.523	98.30%	99.20%
EpiC 3	*Ptgs2*	1.27E-12	1.88E-08	0.505	50.60%	33.70%
EpiC 3	*Itgav*	1.92E-12	2.84E-08	0.541	63.40%	52.80%
EpiC 3	*Nppb*	5.86E-12	8.68E-08	0.526	37.10%	21.60%
EpiC 3	*Ccl20*	8.56E-12	1.27E-07	0.645	15.90%	5.70%
EpiC 3	*Gadd45b*	3.68E-11	5.46E-07	0.518	53.70%	41.00%
EpiC 3	*1810011O10Rik*	1.82E-10	2.70E-06	0.490	72.40%	58.70%
EpiC 3	*Tacstd2*	4.92E-10	7.29E-06	0.556	78.40%	74.60%
EpiC 3	*Malat1*	1.21E-09	1.79E-05	0.877	99.00%	97.10%
EpiC 3	*Bag3*	6.75E-09	1.00E-04	0.505	79.10%	75.20%
EpiC 3	*Cxcl2*	1.16E-08	1.72E-04	0.641	24.70%	13.60%
EpiC 4	*Ephx3*	5.17E-111	7.66E-107	0.546	40.90%	0.90%
EpiC 4	*Slc5a1*	6.10E-89	9.04E-85	0.806	50.00%	3.20%
EpiC 4	*Rhov*	2.06E-80	3.06E-76	0.530	59.10%	5.70%
EpiC 4	*Serpinb3a*	3.97E-80	5.88E-76	1.001	31.80%	0.90%
EpiC 4	*Serpinb12*	1.35E-78	2.00E-74	0.820	33.60%	1.20%
EpiC 4	*Zfp750*	1.68E-73	2.49E-69	0.699	61.80%	7.40%
EpiC 4	*Pvrl4*	1.89E-73	2.81E-69	0.697	64.50%	8.30%
EpiC 4	*Grhl3*	6.72E-61	9.96E-57	0.850	62.70%	9.60%
EpiC 4	*Krt4*	7.95E-57	1.18E-52	3.810	89.10%	31.80%
EpiC 4	*Ly6d*	3.52E-56	5.22E-52	2.599	98.20%	48.50%
EpiC 4	*Krt13*	1.03E-55	1.53E-51	3.775	97.30%	51.60%
EpiC 4	*Sprr2d*	4.68E-55	6.94E-51	3.037	48.20%	6.00%
EpiC 4	*Gsdmc*	6.71E-55	9.94E-51	0.586	40.90%	4.00%

Cluster	Genes	*P* value	FDR	log2(avg diff)	% cells 1	% cells 2
EpiC 4	*Aldh3b2*	7.91E-53	1.17E-48	1.138	84.50%	23.30%
EpiC 4	*43713*	8.95E-52	1.33E-47	0.612	59.10%	10.30%
EpiC 4	*Pdzk1ip1*	1.58E-51	2.34E-47	1.749	94.50%	39.20%
EpiC 4	*Serpinb1a*	1.97E-50	2.93E-46	0.997	86.40%	25.20%
EpiC 4	*Sprr2f*	2.32E-50	3.44E-46	2.047	44.50%	5.40%
EpiC 4	*Psapl1*	8.42E-50	1.25E-45	0.494	44.50%	5.60%
EpiC 4	*Ly6g6c*	9.97E-50	1.48E-45	1.308	32.70%	2.60%
EpiC 4	*Sult2b1*	2.83E-49	4.19E-45	1.405	93.60%	33.30%
EpiC 4	*Clic3*	3.76E-49	5.57E-45	0.746	54.50%	9.20%
EpiC 4	*Fbp2*	6.19E-49	9.17E-45	0.556	60.00%	10.80%
EpiC 4	*Rab25*	2.47E-48	3.66E-44	1.541	98.20%	75.70%
EpiC 4	*Fabp5*	5.89E-48	8.72E-44	2.792	99.10%	89.60%
EpiC 4	*Plbd1*	2.36E-46	3.50E-42	0.840	86.40%	28.30%
EpiC 4	*Capns2*	5.28E-46	7.82E-42	0.994	79.10%	23.10%
EpiC 4	*Pinlyp*	5.30E-46	7.86E-42	1.068	63.60%	14.50%
EpiC 4	*Spink5*	3.41E-45	5.05E-41	1.236	65.50%	13.60%
EpiC 4	*Calm4*	4.65E-45	6.88E-41	2.772	80.90%	27.00%
EpiC 4	*Dmkn*	1.25E-44	1.85E-40	1.817	99.10%	83.50%
EpiC 4	*Lypd3*	4.26E-44	6.31E-40	1.619	98.20%	56.10%
EpiC 4	*Pdlim2*	6.16E-44	9.13E-40	0.658	58.20%	12.00%
EpiC 4	*4833423E24Rik*	3.16E-43	4.69E-39	0.518	59.10%	11.80%
EpiC 4	*Mall*	1.94E-42	2.88E-38	0.846	76.40%	22.60%
EpiC 4	*Fam25c*	3.05E-42	4.52E-38	0.794	45.50%	7.40%
EpiC 4	*Rbp2*	5.90E-41	8.75E-37	0.918	42.70%	6.60%
EpiC 4	*Cst6*	2.01E-40	2.98E-36	0.535	33.60%	3.90%
EpiC 4	*S100a16*	1.33E-39	1.97E-35	1.021	100.00%	95.60%
EpiC 4	*Krt32*	3.50E-39	5.19E-35	0.514	43.60%	6.80%
EpiC 4	*Sprr2h*	2.89E-38	4.29E-34	1.334	51.80%	10.10%
EpiC 4	*Krt6b*	1.90E-37	2.81E-33	3.354	80.90%	33.80%
EpiC 4	*S100a14*	3.68E-37	5.45E-33	0.931	100.00%	92.20%
EpiC 4	*Sbsn*	2.97E-36	4.41E-32	2.085	91.80%	48.60%
EpiC 4	*Dsc2*	6.45E-36	9.56E-32	1.217	83.60%	36.10%
EpiC 4	*Tgm1*	4.48E-35	6.63E-31	1.128	84.50%	30.20%
EpiC 4	*Hebp2*	4.87E-35	7.21E-31	0.649	65.50%	19.60%
EpiC 4	*Gltp*	9.95E-35	1.47E-30	1.210	97.30%	75.90%
EpiC 4	*Pycard*	1.51E-34	2.24E-30	1.116	93.60%	63.00%
EpiC 4	*Mt4*	8.96E-34	1.33E-29	2.670	86.40%	48.80%
EpiC 4	*Hsd17b2*	1.56E-33	2.31E-29	0.576	60.00%	15.40%
EpiC 4	*Tgm3*	3.14E-33	4.65E-29	2.110	60.00%	17.60%
EpiC 4	*Jup*	5.41E-33	8.01E-29	1.090	100.00%	91.20%
EpiC 4	*Klk12*	1.01E-32	1.50E-28	0.624	23.60%	2.20%

Cluster	Genes	*P* value	FDR	log2(avg diff)	% cells 1	% cells 2
EpiC 4	*Cysrt1*	4.42E-32	6.55E-28	0.773	52.70%	12.90%
EpiC 4	*Lgals3*	1.67E-31	2.47E-27	1.317	96.40%	74.70%
EpiC 4	*Klk13*	1.77E-31	2.63E-27	0.991	34.50%	5.30%
EpiC 4	*Ceacam1*	3.26E-31	4.83E-27	0.552	30.00%	3.90%
EpiC 4	*Sprr1a*	7.51E-31	1.11E-26	2.817	54.50%	14.60%
EpiC 4	*Gsta1*	1.54E-30	2.28E-26	1.266	78.20%	28.30%
EpiC 4	*Gsta2*	5.82E-30	8.63E-26	0.733	65.50%	19.10%
EpiC 4	*Rab11a*	7.57E-30	1.12E-25	0.918	100.00%	87.20%
EpiC 4	*Tmprss4*	9.67E-30	1.43E-25	0.966	88.20%	44.00%
EpiC 4	*Endou*	1.19E-29	1.76E-25	0.709	31.80%	4.90%
EpiC 4	*Asprv1*	2.27E-29	3.37E-25	0.868	38.20%	7.00%
EpiC 4	*Gm94*	6.10E-29	9.04E-25	1.914	66.40%	22.70%
EpiC 4	*Chchd10*	2.34E-28	3.46E-24	0.923	87.30%	48.40%
EpiC 4	*Sprr2i*	2.43E-28	3.59E-24	1.400	21.80%	2.20%
EpiC 4	*Evpl*	2.90E-28	4.30E-24	0.674	60.00%	18.60%
EpiC 4	*Perp*	3.93E-28	5.83E-24	0.894	100.00%	99.80%
EpiC 4	*Rnh1*	1.13E-27	1.68E-23	0.789	98.20%	87.10%
EpiC 4	*Dgat2*	2.83E-27	4.19E-23	0.679	65.50%	25.50%
EpiC 4	*Gsta4*	9.99E-26	1.48E-21	0.945	100.00%	97.80%
EpiC 4	*Cldn4*	1.14E-25	1.69E-21	1.669	43.60%	10.30%
EpiC 4	*Trim29*	1.54E-25	2.28E-21	0.801	98.20%	87.00%
EpiC 4	*Pkp1*	1.58E-25	2.34E-21	0.881	98.20%	82.80%
EpiC 4	*Mpzl2*	3.46E-25	5.13E-21	0.653	84.50%	46.80%
EpiC 4	*1810037I17Rik*	4.30E-25	6.37E-21	0.783	99.10%	95.30%
EpiC 4	*Rnase2b*	5.59E-25	8.28E-21	0.659	30.90%	5.50%
EpiC 4	*Ovol1*	1.21E-24	1.79E-20	0.902	66.40%	24.20%
EpiC 4	*Prr13*	1.31E-24	1.95E-20	0.903	98.20%	85.30%
EpiC 4	*Wfdc18*	7.27E-24	1.08E-19	1.535	24.50%	3.50%
EpiC 4	*Crct1*	7.29E-24	1.08E-19	1.785	33.60%	6.70%
EpiC 4	*Klk11*	2.14E-23	3.17E-19	0.600	72.70%	30.70%
EpiC 4	*Sfn*	2.17E-23	3.22E-19	0.855	100.00%	99.60%
EpiC 4	*Dynap*	3.30E-23	4.88E-19	0.892	59.10%	20.40%
EpiC 4	*Ppl*	3.86E-23	5.72E-19	0.500	44.50%	11.70%
EpiC 4	*Krtdap*	4.56E-23	6.76E-19	2.524	84.50%	61.70%
EpiC 4	*Mien1*	8.60E-23	1.27E-18	0.732	94.50%	76.80%
EpiC 4	*Ablim1*	1.35E-22	2.01E-18	0.501	64.50%	24.10%
EpiC 4	*Calml3*	1.59E-22	2.36E-18	1.630	52.70%	17.90%
EpiC 4	*Prdx5*	3.00E-22	4.45E-18	1.381	96.40%	88.90%
EpiC 4	*Teddm3*	3.50E-22	5.19E-18	0.557	54.50%	17.00%
EpiC 4	*Elf3*	3.65E-22	5.41E-18	1.009	82.70%	42.60%
EpiC 4	*Cnfn*	4.07E-22	6.03E-18	2.149	20.90%	2.80%

Cluster	Genes	*P* value	FDR	log2(avg diff)	% cells 1	% cells 2
EpiC 4	*Rab3d*	1.15E-21	1.71E-17	0.487	81.80%	38.90%
EpiC 4	*Ahnak*	1.16E-21	1.72E-17	0.954	98.20%	86.40%
EpiC 4	*Gk*	2.44E-20	3.61E-16	0.559	73.60%	35.70%
EpiC 4	*Sprr2b*	2.59E-20	3.83E-16	0.849	13.60%	1.20%
EpiC 4	*Crabp2*	4.64E-20	6.88E-16	0.788	52.70%	19.00%
EpiC 4	*Ces1h*	4.66E-19	6.90E-15	0.487	69.10%	29.00%
EpiC 4	*Hk2*	6.84E-19	1.01E-14	0.607	73.60%	35.70%
EpiC 4	*Sprr2a3*	1.26E-18	1.87E-14	0.827	45.50%	14.90%
EpiC 4	*S100a9*	1.55E-18	2.30E-14	2.465	71.80%	42.30%
EpiC 4	*Krt16*	1.66E-18	2.46E-14	2.157	87.30%	57.70%
EpiC 4	*Sprr1b*	2.54E-18	3.76E-14	1.800	46.40%	16.30%
EpiC 4	*Cdkn1a*	2.71E-18	4.02E-14	0.816	94.50%	62.60%
EpiC 4	*Glrx*	3.36E-18	4.98E-14	0.848	67.30%	32.80%
EpiC 4	*Chil4*	3.46E-18	5.13E-14	1.819	42.70%	12.60%
EpiC 4	*Ctnnbip1*	1.97E-17	2.92E-13	0.517	80.00%	48.20%
EpiC 4	*Eif6*	3.17E-17	4.70E-13	0.566	98.20%	95.20%
EpiC 4	*Gjb2*	5.95E-17	8.82E-13	0.570	80.00%	43.20%
EpiC 4	*Krt6a*	7.60E-17	1.13E-12	1.202	98.20%	91.30%
EpiC 4	*Ak2*	1.40E-16	2.08E-12	0.514	98.20%	81.50%
EpiC 4	*Anxa1*	1.71E-16	2.53E-12	0.893	100.00%	97.90%
EpiC 4	*Krt23*	1.85E-16	2.74E-12	0.898	18.20%	2.90%
EpiC 4	*Gsto1*	2.89E-16	4.28E-12	1.166	100.00%	97.60%
EpiC 4	*Slc9a3r1*	3.75E-16	5.55E-12	0.703	83.60%	56.70%
EpiC 4	*Dstn*	3.97E-16	5.88E-12	0.510	100.00%	97.90%
EpiC 4	*Ppp1cb*	1.62E-15	2.40E-11	0.599	92.70%	76.10%
EpiC 4	*Slpi*	1.64E-15	2.43E-11	1.749	45.50%	16.80%
EpiC 4	*Vat1*	1.83E-15	2.71E-11	0.567	81.80%	52.10%
EpiC 4	*Ralbp1*	1.83E-15	2.71E-11	0.640	98.20%	88.90%
EpiC 4	*2610528A11Rik*	2.40E-15	3.55E-11	0.984	72.70%	38.60%
EpiC 4	*Dsp*	3.17E-15	4.69E-11	0.771	100.00%	96.00%
EpiC 4	*Tubb2a*	3.28E-15	4.86E-11	0.748	69.10%	33.80%
EpiC 4	*Sprr2e*	4.33E-15	6.42E-11	0.585	12.70%	1.50%
EpiC 4	*Pdzk1*	4.96E-15	7.35E-11	1.146	29.10%	8.10%
EpiC 4	*Idi1*	5.79E-15	8.57E-11	0.502	80.90%	42.00%
EpiC 4	*Rdh12*	5.85E-15	8.67E-11	0.702	56.40%	26.50%
EpiC 4	*Vsig8*	6.17E-15	9.14E-11	0.692	68.20%	35.10%
EpiC 4	*Shroom3*	1.48E-14	2.19E-10	0.538	66.40%	33.70%
EpiC 4	*S100a8*	1.63E-14	2.41E-10	2.275	74.50%	54.80%
EpiC 4	*Idh1*	2.19E-14	3.24E-10	0.596	84.50%	63.20%
EpiC 4	*Ces1b*	2.34E-14	3.46E-10	0.513	56.40%	24.20%
EpiC 4	*Cstb*	3.89E-14	5.77E-10	0.516	100.00%	99.20%

Cluster	Genes	*P* value	FDR	log2(avg diff)	% cells 1	% cells 2
EpiC 4	*Uox*	5.73E-14	8.49E-10	0.654	21.80%	4.70%
EpiC 4	*Csta1*	1.52E-12	2.25E-08	0.682	73.60%	41.90%
EpiC 4	*Gpx2*	2.17E-12	3.22E-08	0.728	96.40%	92.20%
EpiC 4	*Klk10*	3.34E-12	4.95E-08	1.558	69.10%	43.60%
EpiC 4	*Dbi*	3.70E-12	5.48E-08	0.680	99.10%	95.80%
EpiC 4	*Chit1*	3.78E-12	5.61E-08	0.612	90.90%	59.80%
EpiC 4	*Plet1*	3.96E-12	5.87E-08	0.819	49.10%	22.20%
EpiC 4	*Sprr2a1*	7.99E-12	1.18E-07	1.163	64.50%	37.20%
EpiC 4	*Mxd1*	1.38E-11	2.05E-07	0.490	48.20%	20.50%
EpiC 4	*Fgfbp1*	1.60E-11	2.37E-07	0.761	81.80%	52.70%
EpiC 4	*Serpinb5*	1.85E-11	2.74E-07	0.532	98.20%	93.40%
EpiC 4	*Rab24*	3.03E-11	4.50E-07	0.562	90.90%	70.80%
EpiC 4	*Dynll1*	5.58E-11	8.27E-07	0.566	100.00%	98.10%
EpiC 4	*Sptssb*	5.62E-11	8.32E-07	0.912	42.70%	18.80%
EpiC 4	*Mgst3*	6.77E-11	1.00E-06	0.624	87.30%	74.70%
EpiC 4	*Tacstd2*	1.06E-10	1.57E-06	0.879	90.00%	74.50%
EpiC 4	*Higd1a*	1.07E-10	1.58E-06	0.725	97.30%	92.30%
EpiC 4	*Klf4*	1.39E-10	2.05E-06	0.705	89.10%	66.50%
EpiC 4	*Fdps*	1.51E-10	2.24E-06	0.703	84.50%	62.60%
EpiC 4	*Lce3c*	1.90E-10	2.81E-06	0.541	14.50%	2.90%
EpiC 4	*Cyp2f2*	5.92E-10	8.77E-06	0.857	36.40%	14.40%
EpiC 4	*Them5*	6.39E-10	9.46E-06	0.706	80.90%	53.20%
EpiC 4	*Ndufa4*	6.82E-10	1.01E-05	0.619	100.00%	99.10%
EpiC 4	*Arg1*	1.03E-09	1.52E-05	1.221	27.30%	9.40%
EpiC 4	*S100a11*	1.47E-09	2.17E-05	0.598	100.00%	99.40%
EpiC 4	*Pim3*	5.46E-09	8.09E-05	0.540	77.30%	56.10%
EpiC 4	*Ptgr1*	7.97E-09	1.18E-04	0.930	78.20%	61.50%
EpiC 4	*Cbr3*	1.13E-08	1.68E-04	0.535	85.50%	64.20%
EpiC 4	*AA467197*	1.83E-08	2.71E-04	0.961	15.50%	4.00%
EpiC 4	*Mal*	2.52E-08	3.73E-04	0.591	23.60%	8.10%
EpiC 4	*Ltf*	3.12E-08	4.62E-04	0.777	29.10%	11.70%
EpiC 4	*Gstp2*	5.03E-08	7.46E-04	0.801	70.00%	52.70%
EpiC 4	*Nccrp1*	5.52E-08	8.18E-04	0.679	5.50%	0.50%
EpiC 4	*Dsg3*	5.60E-08	8.30E-04	0.595	81.80%	58.30%
EpiC 4	*Prdm1*	6.04E-08	8.95E-04	0.499	30.90%	13.00%
EpiC 5	*Mmp13*	6.57E-79	9.73E-75	3.398	37.60%	2.90%
EpiC 5	*Krt17*	3.78E-77	5.60E-73	2.354	99.50%	73.20%
EpiC 5	*S100a11*	6.72E-71	9.96E-67	1.223	100.00%	99.40%
EpiC 5	*Anxa2*	7.88E-71	1.17E-66	1.510	100.00%	98.30%
EpiC 5	*Slc16a3*	1.22E-66	1.80E-62	1.245	68.10%	18.70%
EpiC 5	*Ndufa4l2*	2.24E-66	3.32E-62	2.123	71.40%	21.90%

Cluster	Genes	*P* value	FDR	log2(avg diff)	% cells 1	% cells 2
EpiC 5	*Inhba*	3.72E-66	5.52E-62	2.422	65.70%	17.40%
EpiC 5	*Car9*	2.99E-64	4.43E-60	0.510	33.80%	3.10%
EpiC 5	*Bnip3*	1.65E-62	2.45E-58	1.940	80.30%	34.90%
EpiC 5	*S100a6*	8.69E-62	1.29E-57	1.399	100.00%	99.40%
EpiC 5	*Tmsb10*	1.86E-59	2.76E-55	1.367	100.00%	97.30%
EpiC 5	*Tpm4*	7.45E-59	1.10E-54	1.021	98.60%	89.70%
EpiC 5	*Anxa1*	6.87E-58	1.02E-53	2.262	100.00%	97.70%
EpiC 5	*Gapdh*	1.20E-57	1.77E-53	1.135	100.00%	99.90%
EpiC 5	*Ndrg1*	6.11E-57	9.05E-53	2.326	85.00%	43.60%
EpiC 5	*Krt6a*	2.29E-56	3.40E-52	1.555	98.60%	90.70%
EpiC 5	*Eno1*	3.09E-56	4.58E-52	1.195	100.00%	98.20%
EpiC 5	*S100a10*	3.68E-55	5.46E-51	1.214	100.00%	98.50%
EpiC 5	*Pgk1*	6.51E-54	9.64E-50	1.328	97.70%	92.70%
EpiC 5	*Tpi1*	2.37E-53	3.52E-49	1.254	99.50%	95.50%
EpiC 5	*Myl6*	6.13E-53	9.09E-49	0.887	100.00%	98.70%
EpiC 5	*Gm5416*	7.57E-53	1.12E-48	1.289	24.90%	1.70%
EpiC 5	*Mmp9*	1.16E-52	1.71E-48	2.020	16.90%	0.30%
EpiC 5	*Fblim1*	2.14E-51	3.17E-47	0.525	58.70%	15.40%
EpiC 5	*Tpm1*	2.96E-51	4.38E-47	1.238	99.10%	90.10%
EpiC 5	*Lama3*	2.96E-51	4.39E-47	1.596	90.60%	56.40%
EpiC 5	*Myl12a*	4.19E-50	6.21E-46	0.851	100.00%	97.30%
EpiC 5	*Actn1*	2.40E-49	3.55E-45	1.102	93.00%	61.40%
EpiC 5	*Pdlim7*	3.57E-49	5.29E-45	0.896	84.50%	46.10%
EpiC 5	*Fhl2*	4.08E-49	6.04E-45	0.852	82.60%	40.40%
EpiC 5	*Prelid2*	5.19E-49	7.68E-45	0.602	50.70%	12.80%
EpiC 5	*Lamc2*	8.93E-48	1.32E-43	1.410	91.10%	57.80%
EpiC 5	*Cav1*	1.80E-47	2.66E-43	1.015	82.60%	43.20%
EpiC 5	*Egln3*	4.14E-47	6.13E-43	1.465	62.40%	22.00%
EpiC 5	*Aldoa*	5.97E-47	8.84E-43	1.013	99.50%	98.30%
EpiC 5	*Prss22*	6.68E-47	9.90E-43	0.587	37.60%	6.40%
EpiC 5	*Itgb6*	1.11E-46	1.65E-42	0.797	58.20%	18.80%
EpiC 5	*Ldha*	2.00E-46	2.96E-42	1.028	99.50%	99.20%
EpiC 5	*Tagln2*	5.16E-45	7.64E-41	1.146	100.00%	98.20%
EpiC 5	*Actb*	6.43E-45	9.53E-41	1.103	100.00%	99.80%
EpiC 5	*Mmp10*	7.32E-45	1.08E-40	1.934	28.60%	3.60%
EpiC 5	*Capg*	1.35E-44	2.00E-40	0.980	98.60%	94.40%
EpiC 5	*Gjb4*	4.00E-44	5.92E-40	0.861	50.70%	13.70%
EpiC 5	*Vegfa*	5.09E-43	7.54E-39	1.088	65.70%	25.80%
EpiC 5	*2200002D01Rik*	6.48E-43	9.60E-39	0.835	94.40%	59.90%
EpiC 5	*Cald1*	9.56E-43	1.42E-38	0.939	89.70%	47.80%
EpiC 5	*Msn*	1.21E-42	1.80E-38	0.813	96.70%	75.90%

Cluster	Genes	*P* value	FDR	log2(avg diff)	% cells 1	% cells 2
EpiC 5	*Higd1a*	1.32E-42	1.96E-38	1.221	98.10%	91.90%
EpiC 5	*Cotl1*	1.56E-42	2.31E-38	0.961	96.20%	78.10%
EpiC 5	*Krt16*	7.11E-42	1.05E-37	1.776	88.30%	55.60%
EpiC 5	*Kctd11*	1.73E-41	2.56E-37	0.743	64.30%	23.50%
EpiC 5	*Tmsb4x*	2.05E-41	3.03E-37	0.890	100.00%	99.30%
EpiC 5	*Fam167a*	2.30E-41	3.41E-37	0.744	72.80%	32.90%
EpiC 5	*Anxa3*	4.41E-41	6.53E-37	0.924	90.10%	64.60%
EpiC 5	*Rbp1*	1.12E-40	1.67E-36	1.052	57.30%	18.30%
EpiC 5	*Gm9844*	1.95E-40	2.88E-36	0.823	86.90%	51.80%
EpiC 5	*Pfn1*	2.44E-40	3.62E-36	0.671	100.00%	99.20%
EpiC 5	*Col18a1*	6.74E-40	9.98E-36	0.652	74.60%	34.00%
EpiC 5	*Tgfbi*	7.49E-40	1.11E-35	1.317	94.80%	77.40%
EpiC 5	*Calm1*	1.09E-39	1.62E-35	0.843	99.50%	98.10%
EpiC 5	*Rhoc*	1.12E-39	1.66E-35	0.688	63.40%	23.50%
EpiC 5	*Aplp2*	1.38E-39	2.05E-35	1.018	93.90%	74.10%
EpiC 5	*Csf3*	1.83E-39	2.71E-35	2.008	23.90%	2.70%
EpiC 5	*Lrrfip1*	9.42E-39	1.40E-34	0.760	83.10%	50.20%
EpiC 5	*Tpm2*	1.10E-38	1.63E-34	0.920	98.10%	93.00%
EpiC 5	*Capns1*	1.86E-38	2.75E-34	0.798	96.20%	90.90%
EpiC 5	*Fam162a*	2.58E-38	3.82E-34	1.294	100.00%	97.70%
EpiC 5	*Itgb1*	3.31E-38	4.90E-34	0.939	98.10%	89.60%
EpiC 5	*Pgam1*	8.96E-38	1.33E-33	0.838	100.00%	97.30%
EpiC 5	*Sfn*	1.40E-37	2.08E-33	0.856	99.50%	99.60%
EpiC 5	*Slco2a1*	2.19E-37	3.24E-33	0.676	53.50%	17.20%
EpiC 5	*Serpinb6a*	5.98E-37	8.87E-33	0.895	97.70%	90.10%
EpiC 5	*Bnip3l*	6.82E-36	1.01E-31	0.851	93.40%	83.50%
EpiC 5	*Prss12*	1.04E-35	1.54E-31	0.564	70.00%	31.00%
EpiC 5	*Pls3*	1.58E-35	2.35E-31	0.909	86.90%	62.30%
EpiC 5	*Cxcl16*	2.43E-35	3.60E-31	0.808	75.10%	37.30%
EpiC 5	*Map7d1*	2.60E-35	3.86E-31	0.823	78.40%	45.80%
EpiC 5	*Rab31*	1.43E-34	2.12E-30	0.493	65.30%	26.90%
EpiC 5	*Acsbg1*	1.47E-34	2.17E-30	0.817	58.20%	23.20%
EpiC 5	*Smox*	1.55E-34	2.30E-30	1.152	72.30%	43.90%
EpiC 5	*Selk*	1.56E-34	2.31E-30	0.721	98.60%	93.40%
EpiC 5	*Txndc17*	2.22E-34	3.29E-30	0.582	100.00%	97.40%
EpiC 5	*S100a14*	3.20E-34	4.75E-30	0.935	97.70%	92.00%
EpiC 5	*Fxyd5*	3.38E-34	5.01E-30	0.801	49.80%	16.10%
EpiC 5	*Ddit4*	6.20E-34	9.19E-30	1.132	91.10%	76.50%
EpiC 5	*Abracl*	1.08E-33	1.60E-29	0.750	97.70%	89.60%
EpiC 5	*Urah*	1.31E-33	1.94E-29	0.966	97.20%	91.80%
EpiC 5	*Krt6b*	1.50E-33	2.22E-29	1.405	69.00%	32.30%

Cluster	Genes	*P* value	FDR	log2(avg diff)	% cells 1	% cells 2
EpiC 5	*Sfr1*	1.82E-33	2.69E-29	0.645	99.50%	95.30%
EpiC 5	*Arpc1b*	2.86E-33	4.24E-29	0.701	98.60%	92.70%
EpiC 5	*Pxdc1*	4.68E-33	6.93E-29	1.117	83.60%	48.70%
EpiC 5	*Dstn*	5.34E-33	7.91E-29	1.052	100.00%	97.80%
EpiC 5	*Sh3glb1*	8.22E-33	1.22E-28	0.629	95.80%	88.50%
EpiC 5	*Cd63*	1.20E-32	1.77E-28	0.563	97.20%	88.10%
EpiC 5	*Tubb3*	1.61E-32	2.38E-28	0.647	41.30%	11.80%
EpiC 5	*Lamb3*	1.82E-32	2.69E-28	1.095	90.60%	71.80%
EpiC 5	*Flnb*	2.27E-31	3.36E-27	0.857	81.20%	53.80%
EpiC 5	*Tnfrsf23*	3.08E-31	4.56E-27	0.597	56.30%	21.60%
EpiC 5	*Cd9*	3.51E-31	5.20E-27	0.642	100.00%	99.30%
EpiC 5	*Odc1*	5.56E-31	8.24E-27	1.760	87.80%	68.10%
EpiC 5	*Pmepa1*	5.67E-31	8.40E-27	0.800	83.10%	56.40%
EpiC 5	*Flrt3*	1.34E-30	1.99E-26	0.650	61.00%	23.60%
EpiC 5	*Cfl1*	1.41E-30	2.10E-26	0.534	100.00%	99.40%
EpiC 5	*Myadm*	1.59E-30	2.36E-26	0.691	75.60%	43.20%
EpiC 5	*Pgf*	3.33E-30	4.93E-26	0.549	16.00%	1.40%
EpiC 5	*Fermt1*	3.40E-30	5.03E-26	1.106	87.30%	69.00%
EpiC 5	*Pkm*	9.21E-30	1.36E-25	0.658	100.00%	99.50%
EpiC 5	*Capzb*	1.04E-29	1.55E-25	0.525	99.10%	93.90%
EpiC 5	*Rala*	3.47E-29	5.15E-25	0.573	94.80%	82.60%
EpiC 5	*Epcam*	6.63E-29	9.82E-25	1.060	89.70%	68.00%
EpiC 5	*Ctsh*	7.36E-29	1.09E-24	0.526	71.40%	39.30%
EpiC 5	*Tspo*	7.91E-29	1.17E-24	0.577	99.50%	96.20%
EpiC 5	*Anxa8*	1.06E-28	1.57E-24	0.843	95.30%	85.30%
EpiC 5	*Tnf*	1.27E-28	1.88E-24	0.746	36.60%	10.10%
EpiC 5	*Hspb1*	1.29E-28	1.91E-24	0.870	100.00%	98.80%
EpiC 5	*Sh3bgrl3*	1.52E-28	2.25E-24	0.730	96.70%	87.80%
EpiC 5	*Cast*	5.80E-28	8.59E-24	0.635	88.70%	75.50%
EpiC 5	*Ndfip1*	5.96E-28	8.84E-24	0.636	96.70%	86.20%
EpiC 5	*Itga5*	7.18E-28	1.06E-23	0.714	46.50%	17.00%
EpiC 5	*Tns4*	7.74E-28	1.15E-23	0.692	91.10%	74.90%
EpiC 5	*Cd44*	1.09E-27	1.61E-23	0.850	95.80%	89.70%
EpiC 5	*Eif4ebp1*	1.69E-27	2.50E-23	0.750	90.10%	75.80%
EpiC 5	*Pfkp*	1.86E-27	2.76E-23	0.655	80.80%	55.20%
EpiC 5	*Lgals3*	3.48E-27	5.15E-23	1.004	93.90%	73.60%
EpiC 5	*Pgk1-rs7*	3.62E-27	5.36E-23	0.575	66.70%	36.60%
EpiC 5	*Arpc5*	9.12E-27	1.35E-22	0.671	97.20%	87.00%
EpiC 5	*Mal*	1.31E-26	1.94E-22	0.872	28.20%	6.40%
EpiC 5	*Col17a1*	1.85E-26	2.74E-22	0.712	97.20%	94.20%
EpiC 5	*Ostf1*	1.90E-26	2.82E-22	0.600	88.70%	77.90%

Cluster	Genes	*P* value	FDR	log2(avg diff)	% cells 1	% cells 2
EpiC 5	*Tes*	3.44E-26	5.10E-22	0.587	83.60%	59.40%
EpiC 5	*Ier3*	1.48E-25	2.19E-21	1.346	93.90%	82.80%
EpiC 5	*Upp1*	1.80E-25	2.67E-21	0.597	57.30%	23.60%
EpiC 5	*Ero1l*	1.91E-25	2.83E-21	1.322	71.40%	45.10%
EpiC 5	*Ppp1r18*	2.50E-25	3.71E-21	0.501	63.80%	32.30%
EpiC 5	*Serpine1*	2.55E-25	3.79E-21	1.280	52.10%	22.60%
EpiC 5	*Tnfrsf12a*	3.73E-25	5.52E-21	0.599	90.10%	64.90%
EpiC 5	*Cap1*	4.95E-25	7.33E-21	0.532	84.50%	65.70%
EpiC 5	*Ehd1*	5.16E-25	7.65E-21	0.691	79.80%	58.20%
EpiC 5	*Slc2a1*	6.93E-25	1.03E-20	0.914	94.80%	92.50%
EpiC 5	*Bsg*	2.02E-24	3.00E-20	0.615	99.10%	97.30%
EpiC 5	*Mxi1*	2.52E-24	3.74E-20	0.621	54.90%	26.10%
EpiC 5	*Capn2*	2.85E-24	4.23E-20	0.578	74.20%	48.50%
EpiC 5	*Fgfbp1*	5.42E-24	8.03E-20	1.275	74.20%	51.80%
EpiC 5	*Tgfa*	5.46E-24	8.08E-20	0.486	62.90%	31.50%
EpiC 5	*Mif*	6.60E-24	9.78E-20	0.508	100.00%	99.20%
EpiC 5	*Pttg1ip*	6.90E-24	1.02E-19	0.632	81.70%	58.10%
EpiC 5	*Alas1*	8.95E-24	1.33E-19	1.044	72.30%	47.00%
EpiC 5	*Fscn1*	1.74E-23	2.58E-19	0.636	94.40%	81.10%
EpiC 5	*Anxa11*	2.41E-23	3.57E-19	0.537	85.90%	65.10%
EpiC 5	*Gng5*	4.79E-23	7.10E-19	0.549	99.50%	98.30%
EpiC 5	*Actr3*	5.99E-23	8.87E-19	0.625	93.40%	89.00%
EpiC 5	*Actg1*	9.59E-23	1.42E-18	0.759	100.00%	99.90%
EpiC 5	*Col7a1*	1.28E-22	1.90E-18	0.634	74.60%	47.10%
EpiC 5	*Clic4*	1.61E-22	2.39E-18	0.674	86.90%	66.90%
EpiC 5	*Anxa5*	1.65E-22	2.45E-18	0.648	98.60%	94.90%
EpiC 5	*BC100530*	1.94E-22	2.88E-18	1.856	57.30%	27.30%
EpiC 5	*Itga6*	2.65E-22	3.93E-18	0.698	94.40%	87.80%
EpiC 5	*Procr*	2.74E-22	4.06E-18	1.265	92.50%	85.40%
EpiC 5	*Pdlim1*	3.11E-22	4.61E-18	0.527	96.20%	91.90%
EpiC 5	*Eno1b*	3.37E-22	5.00E-18	0.510	76.10%	49.60%
EpiC 5	*Pkp1*	4.60E-22	6.81E-18	0.528	93.90%	82.40%
EpiC 5	*Plet1*	5.16E-22	7.65E-18	1.555	46.90%	20.70%
EpiC 5	*Hif1a*	6.87E-22	1.02E-17	0.633	96.70%	92.40%
EpiC 5	*Myo1b*	9.76E-22	1.45E-17	0.580	78.40%	50.40%
EpiC 5	*Crip1*	1.53E-21	2.26E-17	1.525	91.10%	77.70%
EpiC 5	*Ywhag*	2.11E-21	3.12E-17	0.558	93.40%	84.30%
EpiC 5	*Serpinb5*	2.49E-21	3.69E-17	0.570	98.60%	93.10%
EpiC 5	*Csta1*	2.66E-21	3.94E-17	1.786	69.00%	40.40%
EpiC 5	*Pfkl*	3.01E-21	4.46E-17	0.605	81.70%	68.10%
EpiC 5	*Bok*	1.00E-20	1.48E-16	0.616	80.30%	60.50%

Cluster	Genes	*P* value	FDR	log2(avg diff)	% cells 1	% cells 2
EpiC 5	*Wdr1*	1.29E-20	1.92E-16	0.558	95.30%	85.70%
EpiC 5	*Ctsl*	1.40E-20	2.08E-16	0.705	95.80%	82.80%
EpiC 5	*Tuba1a*	1.55E-20	2.30E-16	0.558	70.00%	38.80%
EpiC 5	*Plin2*	3.22E-20	4.77E-16	0.858	67.60%	43.30%
EpiC 5	*Fkbp1a*	5.81E-20	8.60E-16	0.541	95.80%	90.30%
EpiC 5	*Pdcl3*	9.27E-20	1.37E-15	0.496	88.70%	72.10%
EpiC 5	*Sult2b1*	1.21E-19	1.79E-15	0.998	58.70%	34.10%
EpiC 5	*Lypd3*	1.24E-19	1.83E-15	0.817	81.70%	55.60%
EpiC 5	*Gjb2*	1.37E-19	2.02E-15	1.475	65.30%	42.80%
EpiC 5	*Nppb*	2.15E-19	3.19E-15	1.622	48.40%	22.20%
EpiC 5	*Cdkn1a*	3.32E-19	4.93E-15	1.011	79.30%	62.50%
EpiC 5	*Zyx*	1.10E-18	1.63E-14	0.588	84.00%	71.50%
EpiC 5	*Cstb*	1.35E-18	2.00E-14	0.650	100.00%	99.20%
EpiC 5	*Clcf1*	5.31E-18	7.86E-14	0.530	34.70%	13.70%
EpiC 5	*Ctnna1*	6.92E-18	1.03E-13	0.487	90.10%	79.10%
EpiC 5	*Cnn2*	7.09E-18	1.05E-13	0.693	77.90%	59.80%
EpiC 5	*Anxa7*	7.49E-18	1.11E-13	0.502	95.30%	86.60%
EpiC 5	*Msmo1*	7.73E-18	1.15E-13	0.561	79.30%	55.20%
EpiC 5	*Flna*	1.48E-17	2.20E-13	0.575	85.40%	73.40%
EpiC 5	*Il1a*	1.59E-17	2.36E-13	0.727	31.00%	11.50%
EpiC 5	*Hilpda*	1.61E-17	2.38E-13	0.940	59.20%	35.70%
EpiC 5	*Itgb4*	2.33E-17	3.46E-13	0.607	95.30%	86.20%
EpiC 5	*Krt14*	4.37E-17	6.48E-13	0.561	100.00%	99.10%
EpiC 5	*Isg20*	5.07E-17	7.51E-13	0.499	44.10%	20.70%
EpiC 5	*Nrg1*	5.57E-17	8.25E-13	0.522	66.20%	39.00%
EpiC 5	*Klf7*	9.57E-17	1.42E-12	0.505	61.00%	38.40%
EpiC 5	*Stfa2*	1.51E-16	2.23E-12	0.793	9.90%	1.10%
EpiC 5	*Clu*	5.53E-16	8.19E-12	1.264	54.90%	30.80%
EpiC 5	*Ptges*	5.57E-16	8.25E-12	0.853	67.60%	50.20%
EpiC 5	*Sqstm1*	6.28E-16	9.30E-12	0.606	95.80%	85.90%
EpiC 5	*Acta2*	6.64E-16	9.84E-12	1.435	46.90%	23.70%
EpiC 5	*Ifi202b*	6.70E-16	9.93E-12	0.924	55.90%	32.20%
EpiC 5	*Sdc1*	8.70E-16	1.29E-11	0.509	97.70%	94.30%
EpiC 5	*Crip2*	8.83E-16	1.31E-11	0.661	94.40%	90.10%
EpiC 5	*Plec*	1.47E-15	2.18E-11	0.514	89.70%	82.20%
EpiC 5	*Efhd2*	2.18E-15	3.23E-11	0.689	72.80%	53.30%
EpiC 5	*Plek2*	2.26E-15	3.35E-11	0.534	87.30%	74.80%
EpiC 5	*Htra1*	2.67E-15	3.96E-11	0.695	81.70%	64.70%
EpiC 5	*Mapk6*	7.36E-15	1.09E-10	0.515	86.90%	75.60%
EpiC 5	*Tgm1*	1.04E-14	1.54E-10	0.922	52.60%	31.00%
EpiC 5	*Arg1*	1.06E-14	1.57E-10	0.776	25.40%	8.40%

Cluster	Genes	*P* value	FDR	log2(avg diff)	% cells 1	% cells 2
EpiC 5	*Plau*	2.07E-14	3.07E-10	0.668	54.50%	31.80%
EpiC 5	*Rap1b*	2.42E-14	3.58E-10	0.520	85.90%	78.00%
EpiC 5	*Mxd1*	3.34E-14	4.94E-10	0.523	40.40%	19.80%
EpiC 5	*Crct1*	4.03E-14	5.98E-10	1.278	21.60%	6.50%
EpiC 5	*Denr*	1.04E-13	1.54E-09	0.771	83.60%	76.20%
EpiC 5	*Myh9*	1.87E-13	2.77E-09	0.559	92.50%	85.70%
EpiC 5	*Sprr2h*	1.95E-13	2.89E-09	1.056	27.70%	10.70%
EpiC 5	*Tubb6*	2.13E-13	3.16E-09	0.639	73.20%	53.20%
EpiC 5	*Ptgs2*	5.93E-13	8.78E-09	0.792	57.70%	35.00%
EpiC 5	*Rhob*	6.94E-13	1.03E-08	0.654	86.90%	73.40%
EpiC 5	*Cxcl3*	1.85E-12	2.74E-08	1.693	17.40%	5.00%
EpiC 5	*Fdps*	3.13E-12	4.64E-08	0.628	77.00%	62.20%
EpiC 5	*Igfbp3*	3.29E-12	4.87E-08	0.592	73.70%	52.20%
EpiC 5	*Sptssa*	6.96E-12	1.03E-07	0.490	90.60%	84.70%
EpiC 5	*Epgn*	3.05E-11	4.52E-07	0.831	31.00%	14.30%
EpiC 5	*Lce3c*	1.08E-10	1.61E-06	0.675	11.30%	2.50%
EpiC 5	*Serpinb2*	1.77E-10	2.62E-06	1.604	64.30%	54.40%
EpiC 5	*Prl2c3*	6.11E-10	9.05E-06	1.026	7.50%	1.20%
EpiC 5	*Prl2c2*	7.85E-10	1.16E-05	0.893	5.60%	0.60%
EpiC 5	*Areg*	1.17E-09	1.74E-05	0.862	54.00%	34.00%
EpiC 5	*Il1rn*	2.03E-09	3.01E-05	0.778	57.70%	43.40%
EpiC 5	*Stfa3*	2.36E-09	3.50E-05	1.053	36.60%	19.90%
EpiC 5	*Phlda1*	2.49E-09	3.69E-05	0.774	84.50%	71.60%
EpiC 5	*Mmp12*	5.78E-09	8.56E-05	0.505	12.70%	3.60%
EpiC 5	*Sprr2e*	8.92E-09	1.32E-04	0.727	7.50%	1.40%
EpiC 5	*Plaur*	1.07E-08	1.58E-04	0.578	51.20%	32.50%
EpiC 5	*Il24*	1.17E-08	1.73E-04	0.979	14.10%	4.60%
EpiC 5	*Hcar2*	1.58E-08	2.33E-04	0.784	77.00%	69.70%
EpiC 5	*S100a8*	5.37E-08	7.95E-04	1.050	70.90%	54.00%
EpiC 5	*S100a9*	6.29E-08	9.32E-04	1.446	59.20%	42.10%
EpiC 5	*Irf6*	6.68E-08	9.90E-04	0.558	70.00%	58.60%
EpiC 6	*Hmga2*	4.20E-266	6.22E-262	1.896	87.60%	2.70%
EpiC 6	*Gap43*	4.07E-215	6.02E-211	1.393	69.00%	1.60%
EpiC 6	*Pde3b*	7.76E-214	1.15E-209	0.620	63.80%	0.70%
EpiC 6	*Disp2*	7.52E-210	1.11E-205	0.608	59.50%	0.30%
EpiC 6	*Gm1045*	6.03E-208	8.93E-204	2.796	89.50%	7.80%
EpiC 6	*Cyba*	1.72E-197	2.56E-193	1.959	94.30%	11.60%
EpiC 6	*Krt18*	9.70E-184	1.44E-179	1.911	87.10%	8.60%
EpiC 6	*Prkg2*	1.79E-174	2.65E-170	1.279	77.10%	6.30%
EpiC 6	*Tm4sf1*	1.90E-172	2.82E-168	2.415	83.80%	8.90%
EpiC 6	*Stmn2*	5.17E-163	7.66E-159	1.397	56.20%	1.60%

Cluster	Genes	*P* value	FDR	log2(avg diff)	% cells 1	% cells 2
EpiC 6	*Ppp1r14a*	3.08E-156	4.56E-152	0.761	61.90%	3.10%
EpiC 6	*Fstl1*	4.75E-149	7.04E-145	1.257	86.20%	12.60%
EpiC 6	*Basp1*	1.60E-148	2.37E-144	0.975	78.60%	8.50%
EpiC 6	*Wnt5a*	8.60E-145	1.27E-140	1.382	88.60%	15.50%
EpiC 6	*Fbln2*	8.78E-145	1.30E-140	1.915	80.50%	10.70%
EpiC 6	*Upp1*	1.59E-135	2.35E-131	1.617	92.40%	18.90%
EpiC 6	*Stra6*	8.65E-135	1.28E-130	0.964	68.60%	6.60%
EpiC 6	*Fkbp10*	1.04E-133	1.55E-129	0.511	55.20%	3.00%
EpiC 6	*Gm42835*	2.31E-133	3.42E-129	0.865	59.00%	3.90%
EpiC 6	*Cnn3*	6.97E-132	1.03E-127	1.172	88.10%	16.40%
EpiC 6	*Timp1*	7.10E-131	1.05E-126	2.569	81.90%	14.70%
EpiC 6	*Slitrk6*	1.81E-130	2.68E-126	1.062	75.20%	10.00%
EpiC 6	*Cdkn2a*	3.57E-128	5.29E-124	1.761	96.70%	26.10%
EpiC 6	*Pthlh*	1.16E-121	1.72E-117	4.819	95.20%	32.80%
EpiC 6	*Ecm1*	5.90E-121	8.74E-117	3.547	99.50%	52.70%
EpiC 6	*Emp3*	2.84E-120	4.21E-116	1.475	80.50%	15.30%
EpiC 6	*Soat1*	8.67E-120	1.29E-115	1.126	74.80%	11.30%
EpiC 6	*Calca*	9.81E-119	1.45E-114	0.899	38.60%	0.70%
EpiC 6	*Cd80*	4.37E-116	6.48E-112	0.874	71.00%	10.30%
EpiC 6	*Antxr2*	3.34E-115	4.95E-111	0.865	76.20%	12.00%
EpiC 6	*Cald1*	6.25E-112	9.26E-108	2.251	99.50%	46.60%
EpiC 6	*Pxdn*	2.71E-110	4.02E-106	0.505	51.00%	3.70%
EpiC 6	*Jam2*	4.46E-110	6.60E-106	0.541	41.90%	1.70%
EpiC 6	*Pcdh7*	6.90E-109	1.02E-104	1.186	88.60%	22.30%
EpiC 6	*Kcnn4*	5.86E-108	8.68E-104	0.978	64.80%	8.50%
EpiC 6	*Adam12*	8.96E-107	1.33E-102	0.528	48.60%	3.30%
EpiC 6	*Rbpms*	2.86E-106	4.24E-102	1.006	91.00%	24.70%
EpiC 6	*Cd63*	3.11E-104	4.61E-100	2.157	100.00%	87.80%
EpiC 6	*Tnfrsf23*	2.10E-103	3.11E-99	1.019	85.20%	17.80%
EpiC 6	*Lamb1*	1.27E-102	1.89E-98	0.706	70.00%	11.20%
EpiC 6	*Ctsl*	1.74E-100	2.58E-96	2.332	100.00%	82.20%
EpiC 6	*Tmsb10*	5.11E-100	7.57E-96	2.380	100.00%	97.30%
EpiC 6	*Rai14*	3.33E-99	4.93E-95	0.730	72.90%	13.70%
EpiC 6	*Aplp2*	3.40E-98	5.04E-94	2.243	99.00%	73.50%
EpiC 6	*Uchl1*	3.71E-95	5.50E-91	0.552	30.50%	0.50%
EpiC 6	*A430105I19Rik*	3.46E-94	5.13E-90	0.513	63.30%	9.30%
EpiC 6	*Fads3*	1.39E-93	2.07E-89	0.709	71.90%	13.50%
EpiC 6	*Cmas*	6.76E-92	1.00E-87	1.898	86.20%	30.80%
EpiC 6	*Arntl2*	1.98E-91	2.93E-87	1.329	79.00%	21.30%
EpiC 6	*Serpinf1*	6.23E-91	9.23E-87	0.584	55.20%	6.80%
EpiC 6	*Tnc*	6.43E-91	9.53E-87	1.588	78.10%	18.40%

Cluster	Genes	*P* value	FDR	log2(avg diff)	% cells 1	% cells 2
EpiC 6	*Flrt3*	1.80E-90	2.67E-86	1.065	83.30%	20.60%
EpiC 6	*Aig1*	6.86E-90	1.02E-85	1.530	96.20%	64.80%
EpiC 6	*S100a6*	8.26E-89	1.22E-84	1.930	100.00%	99.40%
EpiC 6	*Stab1*	8.41E-89	1.25E-84	0.688	67.60%	11.90%
EpiC 6	*Tuba1a*	1.26E-86	1.87E-82	1.648	90.50%	36.00%
EpiC 6	*Gm9844*	1.67E-86	2.48E-82	1.363	96.20%	50.60%
EpiC 6	*Ggct*	1.82E-86	2.69E-82	1.159	76.20%	21.00%
EpiC 6	*Golt1b*	3.65E-86	5.40E-82	1.860	90.00%	41.10%
EpiC 6	*Cd9*	1.63E-85	2.41E-81	1.375	100.00%	99.30%
EpiC 6	*Sdc2*	6.33E-85	9.38E-81	0.938	81.40%	25.20%
EpiC 6	*Oaf*	1.36E-84	2.02E-80	1.100	81.90%	23.40%
EpiC 6	*Actn1*	7.33E-84	1.09E-79	1.455	97.60%	60.90%
EpiC 6	*Gm12603*	8.70E-84	1.29E-79	0.783	77.10%	17.70%
EpiC 6	*Itga3*	5.33E-82	7.89E-78	1.558	93.30%	53.90%
EpiC 6	*Lamc2*	1.16E-81	1.71E-77	2.364	95.20%	57.30%
EpiC 6	*Lyrm5*	1.60E-81	2.37E-77	1.062	77.60%	22.30%
EpiC 6	*App*	2.26E-81	3.35E-77	1.506	99.00%	90.90%
EpiC 6	*Tgfb1*	4.14E-81	6.14E-77	1.213	95.20%	54.80%
EpiC 6	*Flna*	5.68E-81	8.41E-77	1.535	98.10%	71.70%
EpiC 6	*Kras*	6.68E-81	9.90E-77	1.833	93.30%	58.00%
EpiC 6	*Fblim1*	8.57E-81	1.27E-76	0.599	70.00%	13.90%
EpiC 6	*Nt5dc2*	8.82E-81	1.31E-76	0.541	61.00%	10.70%
EpiC 6	*Runx1*	2.75E-80	4.08E-76	1.106	95.20%	38.20%
EpiC 6	*Mtap*	1.28E-79	1.90E-75	1.122	93.80%	47.00%
EpiC 6	*Tnfrsf12a*	3.37E-79	4.99E-75	1.579	98.10%	63.90%
EpiC 6	*Teddm3*	1.08E-78	1.60E-74	0.744	65.20%	13.10%
EpiC 6	*Inhba*	1.26E-77	1.86E-73	0.629	76.20%	16.10%
EpiC 6	*Klk8*	1.35E-77	2.00E-73	1.248	77.60%	22.40%
EpiC 6	*Tnnt2*	4.01E-77	5.93E-73	0.500	52.90%	7.70%
EpiC 6	*Slc7a8*	5.91E-77	8.76E-73	0.608	69.00%	14.40%
EpiC 6	*Rhoc*	6.67E-77	9.88E-73	0.664	80.00%	21.30%
EpiC 6	*Hacd1*	7.58E-77	1.12E-72	0.737	83.30%	25.50%
EpiC 6	*Glipr1*	9.55E-76	1.42E-71	0.534	60.50%	11.40%
EpiC 6	*Xirp2*	1.45E-75	2.14E-71	0.944	30.50%	1.40%
EpiC 6	*Porcn*	3.11E-75	4.60E-71	0.786	72.90%	19.50%
EpiC 6	*Hmga1*	3.18E-75	4.71E-71	1.786	95.20%	79.00%
EpiC 6	*Nrg1*	1.34E-74	1.98E-70	1.191	89.50%	35.80%
EpiC 6	*Npc2*	2.88E-74	4.27E-70	1.031	98.60%	90.60%
EpiC 6	*Itgb1*	5.42E-74	8.03E-70	1.409	99.00%	89.50%
EpiC 6	*Fxyd5*	6.45E-74	9.55E-70	0.691	66.70%	13.80%
EpiC 6	*Limd2*	9.30E-74	1.38E-69	0.698	71.90%	19.50%

Cluster	Genes	*P* value	FDR	log2(avg diff)	% cells 1	% cells 2
EpiC 6	*Lama3*	3.73E-73	5.53E-69	1.906	94.30%	56.00%
EpiC 6	*Cd55*	6.83E-73	1.01E-68	0.540	62.90%	12.60%
EpiC 6	*2200002D01Rik*	9.71E-73	1.44E-68	1.234	96.20%	59.70%
EpiC 6	*Gsr*	7.47E-72	1.11E-67	1.196	96.70%	60.10%
EpiC 6	*Snhg18*	1.33E-71	1.98E-67	0.515	68.10%	15.50%
EpiC 6	*Klhl42*	3.46E-71	5.13E-67	0.842	59.50%	12.50%
EpiC 6	*Spp1*	6.45E-71	9.56E-67	0.801	27.10%	1.00%
EpiC 6	*Ccnd1*	1.68E-70	2.49E-66	1.527	95.70%	77.60%
EpiC 6	*Msn*	8.69E-70	1.29E-65	1.166	99.00%	75.60%
EpiC 6	*Ctsz*	3.64E-69	5.39E-65	0.912	93.30%	55.20%
EpiC 6	*Gnas*	1.42E-68	2.10E-64	0.810	100.00%	97.70%
EpiC 6	*Tmem167*	3.65E-68	5.41E-64	0.924	97.60%	81.10%
EpiC 6	*Ppfibp1*	7.39E-68	1.10E-63	1.737	84.30%	39.40%
EpiC 6	*Ppib*	1.04E-67	1.54E-63	0.946	100.00%	97.90%
EpiC 6	*Man1a*	1.60E-67	2.37E-63	0.682	78.10%	24.80%
EpiC 6	*Itga6*	7.13E-67	1.06E-62	1.427	99.00%	87.10%
EpiC 6	*Rras2*	1.42E-66	2.11E-62	0.787	79.50%	27.00%
EpiC 6	*Pls3*	1.68E-66	2.50E-62	1.109	95.70%	61.10%
EpiC 6	*Cpe*	2.27E-66	3.37E-62	1.240	98.60%	80.30%
EpiC 6	*Zdhhc12*	4.71E-66	6.98E-62	0.610	74.30%	23.90%
EpiC 6	*Rcn1*	6.20E-66	9.18E-62	0.984	95.20%	62.70%
EpiC 6	*Pla2g7*	4.00E-65	5.93E-61	0.635	56.20%	10.90%
EpiC 6	*Mlec*	9.52E-65	1.41E-60	1.049	94.30%	65.70%
EpiC 6	*Lgals1*	1.75E-64	2.59E-60	2.122	72.90%	25.20%
EpiC 6	*Myl12a*	2.35E-64	3.49E-60	0.893	100.00%	97.30%
EpiC 6	*Fkbp1a*	3.27E-64	4.84E-60	0.896	99.50%	89.80%
EpiC 6	*Cotl1*	7.27E-64	1.08E-59	1.160	98.60%	77.80%
EpiC 6	*Spcs1*	9.76E-64	1.45E-59	0.810	99.50%	95.10%
EpiC 6	*Itgb4*	1.75E-63	2.59E-59	1.370	98.10%	85.80%
EpiC 6	*Cldn8*	2.20E-63	3.26E-59	0.882	63.80%	16.40%
EpiC 6	*Nppc*	3.57E-63	5.30E-59	0.927	43.80%	6.20%
EpiC 6	*Plekha5*	1.35E-62	1.99E-58	0.940	73.80%	26.20%
EpiC 6	*Serpine2*	2.53E-62	3.74E-58	1.097	89.00%	41.10%
EpiC 6	*Krtcap2*	2.53E-62	3.75E-58	1.007	98.10%	89.60%
EpiC 6	*Arl4c*	5.09E-62	7.54E-58	0.791	82.40%	30.30%
EpiC 6	*Orai2*	8.62E-62	1.28E-57	0.621	71.90%	23.00%
EpiC 6	*Pdpn*	1.50E-61	2.22E-57	1.266	85.20%	39.20%
EpiC 6	*U90926*	1.57E-61	2.32E-57	0.636	26.20%	1.40%
EpiC 6	*Cmtm3*	1.58E-61	2.34E-57	0.511	66.20%	17.10%
EpiC 6	*Med21*	1.98E-61	2.94E-57	2.166	95.20%	79.60%
EpiC 6	*Wipi1*	2.04E-61	3.02E-57	0.603	75.70%	24.60%

Cluster	Genes	*P* value	FDR	log2(avg diff)	% cells 1	% cells 2
EpiC 6	*Areg*	7.74E-61	1.15E-56	2.545	79.00%	30.60%
EpiC 6	*Bsg*	1.32E-60	1.96E-56	0.853	99.50%	97.30%
EpiC 6	*Rab31*	9.23E-60	1.37E-55	0.531	78.60%	25.20%
EpiC 6	*Cdk6*	1.04E-59	1.54E-55	0.826	87.10%	42.60%
EpiC 6	*Rrbp1*	3.30E-59	4.89E-55	0.932	97.10%	80.70%
EpiC 6	*Ppp1r14c*	4.98E-59	7.37E-55	0.743	83.80%	33.00%
EpiC 6	*Fgfr1op2*	6.16E-59	9.12E-55	1.929	94.80%	76.30%
EpiC 6	*Ptprz1*	7.65E-59	1.13E-54	1.204	78.60%	34.50%
EpiC 6	*Jag1*	9.58E-59	1.42E-54	0.994	87.60%	44.40%
EpiC 6	*Nceh1*	1.30E-58	1.93E-54	0.550	70.00%	20.20%
EpiC 6	*Asap2*	1.37E-58	2.02E-54	0.548	71.90%	21.90%
EpiC 6	*Vim*	1.80E-58	2.66E-54	1.909	70.50%	25.10%
EpiC 6	*Flrt2*	1.82E-58	2.69E-54	0.698	66.70%	18.50%
EpiC 6	*Tspan3*	2.85E-58	4.22E-54	1.027	98.60%	82.40%
EpiC 6	*Calcb*	4.85E-58	7.18E-54	0.931	29.50%	2.30%
EpiC 6	*Dnajc15*	6.02E-58	8.92E-54	1.147	91.40%	71.70%
EpiC 6	*Tes*	1.30E-57	1.93E-53	0.790	94.30%	58.00%
EpiC 6	*Ackr3*	3.13E-57	4.64E-53	0.676	70.00%	20.80%
EpiC 6	*Dusp6*	4.77E-57	7.06E-53	0.966	88.60%	42.00%
EpiC 6	*Ssr3*	1.09E-56	1.61E-52	0.946	99.00%	83.60%
EpiC 6	*Dap*	8.95E-56	1.33E-51	0.923	88.10%	52.10%
EpiC 6	*Fscn1*	9.48E-56	1.40E-51	1.016	97.10%	80.80%
EpiC 6	*Slc16a11*	1.39E-55	2.06E-51	0.808	78.10%	29.20%
EpiC 6	*Dad1*	2.07E-55	3.06E-51	0.747	99.00%	94.40%
EpiC 6	*Spsb1*	4.34E-55	6.42E-51	0.566	68.10%	19.90%
EpiC 6	*Arpc1b*	9.77E-55	1.45E-50	0.843	99.50%	92.60%
EpiC 6	*Calu*	1.00E-54	1.49E-50	0.871	91.00%	54.50%
EpiC 6	*Pgam1*	1.16E-54	1.71E-50	0.896	99.50%	97.40%
EpiC 6	*Vmp1*	1.46E-54	2.16E-50	0.791	98.60%	76.80%
EpiC 6	*Sparc*	1.85E-54	2.74E-50	1.750	75.20%	33.80%
EpiC 6	*Crtap*	5.64E-54	8.35E-50	0.903	89.00%	57.20%
EpiC 6	*Col18a1*	6.32E-54	9.36E-50	0.723	83.30%	32.90%
EpiC 6	*Ppp1r18*	1.97E-53	2.91E-49	0.549	80.00%	30.20%
EpiC 6	*Tmsb4x*	4.70E-53	6.97E-49	1.017	100.00%	99.30%
EpiC 6	*Ctsd*	1.03E-52	1.52E-48	1.038	99.50%	96.40%
EpiC 6	*Peak1*	1.17E-52	1.73E-48	0.528	61.40%	17.10%
EpiC 6	*Fat1*	1.22E-52	1.80E-48	0.859	85.70%	41.80%
EpiC 6	*Slc20a1*	1.31E-52	1.94E-48	0.636	80.50%	30.50%
EpiC 6	*Leprotl1*	3.15E-52	4.66E-48	0.715	93.80%	66.00%
EpiC 6	*Pmepa1*	4.85E-52	7.19E-48	0.933	90.50%	55.40%
EpiC 6	*Etnk1*	1.75E-51	2.59E-47	1.049	73.80%	31.50%

Cluster	Genes	*P* value	FDR	log2(avg diff)	% cells 1	% cells 2
EpiC 6	*Pdlim7*	6.63E-51	9.82E-47	0.637	90.50%	45.40%
EpiC 6	*Alcam*	8.11E-51	1.20E-46	0.516	61.00%	16.20%
EpiC 6	*Eno1*	8.13E-51	1.20E-46	0.815	100.00%	98.20%
EpiC 6	*Fth1*	8.97E-51	1.33E-46	0.861	100.00%	99.70%
EpiC 6	*Mapk6*	9.27E-51	1.37E-46	0.904	98.60%	74.00%
EpiC 6	*Epha2*	2.03E-50	3.01E-46	0.832	71.00%	25.60%
EpiC 6	*Acsbg1*	3.06E-50	4.53E-46	0.541	70.50%	21.60%
EpiC 6	*Pdia3*	3.21E-50	4.75E-46	0.828	100.00%	97.10%
EpiC 6	*Serpinb6a*	1.96E-49	2.90E-45	0.792	99.00%	89.90%
EpiC 6	*Mmp14*	2.93E-49	4.34E-45	0.817	95.70%	65.60%
EpiC 6	*Txndc17*	3.51E-49	5.20E-45	0.717	100.00%	97.40%
EpiC 6	*Endod1*	3.52E-49	5.22E-45	0.583	69.50%	24.70%
EpiC 6	*Calr*	7.56E-49	1.12E-44	0.899	99.00%	93.10%
EpiC 6	*Zwint*	1.16E-48	1.72E-44	0.693	92.40%	58.90%
EpiC 6	*Nradd*	1.41E-48	2.08E-44	0.845	87.10%	54.50%
EpiC 6	*Slc48a1*	2.69E-48	3.99E-44	0.798	89.00%	58.50%
EpiC 6	*Tspan31*	3.30E-48	4.90E-44	0.708	93.80%	71.40%
EpiC 6	*Csrp2*	4.00E-48	5.93E-44	0.543	66.20%	22.20%
EpiC 6	*Krt17*	5.08E-48	7.52E-44	1.120	99.00%	73.30%
EpiC 6	*Krt42*	6.02E-48	8.93E-44	0.682	31.00%	3.80%
EpiC 6	*Tmprss11b*	1.14E-47	1.69E-43	0.622	39.00%	6.90%
EpiC 6	*Ereg*	1.24E-47	1.84E-43	0.658	47.10%	10.00%
EpiC 6	*Nptn*	2.50E-47	3.70E-43	0.715	97.10%	72.80%
EpiC 6	*Hsp90b1*	4.91E-47	7.27E-43	1.081	100.00%	95.00%
EpiC 6	*Lamb3*	4.92E-47	7.29E-43	1.713	89.00%	72.10%
EpiC 6	*Plaur*	8.23E-47	1.22E-42	0.780	76.20%	29.20%
EpiC 6	*Jkamp*	1.01E-46	1.50E-42	0.545	86.20%	42.90%
EpiC 6	*Hsd17b12*	2.92E-46	4.33E-42	0.693	92.40%	69.50%
EpiC 6	*Manf*	8.65E-46	1.28E-41	1.093	97.60%	89.50%
EpiC 6	*Mlf2*	1.05E-45	1.56E-41	0.683	99.00%	93.30%
EpiC 6	*Atp2b1*	1.58E-45	2.34E-41	0.845	93.80%	62.00%
EpiC 6	*Chchd7*	2.02E-45	2.99E-41	0.611	85.70%	47.70%
EpiC 6	*Axl*	2.26E-45	3.35E-41	0.524	50.50%	12.80%
EpiC 6	*Galnt1*	4.16E-45	6.17E-41	0.754	84.80%	44.80%
EpiC 6	*Ndfip1*	4.27E-45	6.33E-41	0.714	98.60%	86.00%
EpiC 6	*Rassf8*	5.83E-45	8.64E-41	0.509	50.50%	13.10%
EpiC 6	*Itm2b*	9.38E-45	1.39E-40	0.681	100.00%	99.60%
EpiC 6	*Capns1*	3.08E-44	4.56E-40	0.659	99.50%	90.50%
EpiC 6	*43723*	3.80E-44	5.62E-40	0.646	98.60%	95.70%
EpiC 6	*Pfn1*	4.89E-44	7.25E-40	0.653	99.50%	99.20%
EpiC 6	*Lman1*	5.38E-44	7.97E-40	0.752	96.20%	74.70%

Cluster	Genes	*P* value	FDR	log2(avg diff)	% cells 1	% cells 2
EpiC 6	*Sfr1*	6.31E-44	9.35E-40	0.672	99.00%	95.30%
EpiC 6	*Aprt*	8.98E-44	1.33E-39	0.761	98.10%	91.70%
EpiC 6	*Cav1*	9.63E-44	1.43E-39	0.599	87.60%	42.60%
EpiC 6	*P3h4*	1.36E-43	2.01E-39	0.633	87.60%	52.10%
EpiC 6	*Tmed7*	1.79E-43	2.66E-39	0.623	93.80%	65.30%
EpiC 6	*Sec11c*	2.97E-43	4.40E-39	0.784	90.50%	63.50%
EpiC 6	*Atp6v0b*	3.45E-43	5.11E-39	0.645	97.60%	86.40%
EpiC 6	*Igsf8*	4.64E-43	6.88E-39	0.722	89.50%	52.70%
EpiC 6	*Hsbp1*	5.84E-43	8.65E-39	0.633	97.10%	88.20%
EpiC 6	*Erp44*	9.26E-43	1.37E-38	0.712	92.90%	74.20%
EpiC 6	*Spint2*	9.99E-43	1.48E-38	0.675	99.50%	95.90%
EpiC 6	*Prkar1a*	1.36E-42	2.01E-38	0.604	99.00%	87.40%
EpiC 6	*B4galt1*	1.79E-42	2.65E-38	0.693	91.00%	52.20%
EpiC 6	*Akr1b3*	2.19E-42	3.24E-38	0.770	96.70%	78.60%
EpiC 6	*Cd81*	2.68E-42	3.97E-38	0.836	98.10%	88.00%
EpiC 6	*Arpc5*	3.04E-42	4.51E-38	0.693	98.60%	86.80%
EpiC 6	*Ltbp4*	7.12E-42	1.05E-37	0.696	85.20%	42.40%
EpiC 6	*Selm*	1.04E-41	1.55E-37	0.603	74.80%	31.90%
EpiC 6	*Serinc1*	1.11E-41	1.64E-37	0.612	91.90%	60.30%
EpiC 6	*Camsap2*	1.45E-41	2.15E-37	0.550	81.90%	38.10%
EpiC 6	*Sdc1*	5.51E-41	8.17E-37	0.848	98.60%	94.20%
EpiC 6	*Timp2*	6.05E-41	8.96E-37	0.634	45.20%	10.60%
EpiC 6	*Sparcl1*	7.25E-41	1.07E-36	0.526	37.60%	7.30%
EpiC 6	*Tomm20*	1.24E-40	1.84E-36	0.651	99.50%	95.10%
EpiC 6	*Plin2*	1.26E-40	1.86E-36	0.708	83.30%	41.20%
EpiC 6	*Gnb1*	1.27E-40	1.89E-36	0.592	98.60%	78.50%
EpiC 6	*Myof*	1.95E-40	2.88E-36	0.732	89.50%	58.70%
EpiC 6	*Surf4*	2.16E-40	3.20E-36	0.707	95.20%	80.40%
EpiC 6	*Dnajc3*	6.74E-40	9.98E-36	0.733	96.20%	77.20%
EpiC 6	*Il24*	7.18E-40	1.06E-35	0.718	25.70%	3.00%
EpiC 6	*Tubb6*	7.87E-40	1.17E-35	0.838	89.00%	51.10%
EpiC 6	*Pold4*	1.33E-39	1.98E-35	0.810	88.60%	58.50%
EpiC 6	*Marcksl1*	2.67E-39	3.96E-35	0.720	86.20%	51.30%
EpiC 6	*P4ha1*	3.01E-39	4.46E-35	0.674	90.50%	54.10%
EpiC 6	*Cfl1*	3.76E-39	5.56E-35	0.583	100.00%	99.40%
EpiC 6	*Ercc1*	6.25E-39	9.26E-35	0.639	81.00%	47.70%
EpiC 6	*Homer3*	6.85E-39	1.01E-34	0.498	73.30%	30.40%
EpiC 6	*Ostc*	8.63E-39	1.28E-34	0.663	95.70%	86.60%
EpiC 6	*Tpm1*	8.65E-39	1.28E-34	0.868	98.10%	90.30%
EpiC 6	*Ext1*	1.02E-38	1.51E-34	0.523	73.80%	32.00%
EpiC 6	*Kdelr2*	1.02E-38	1.52E-34	0.738	94.30%	80.50%

Cluster	Genes	*P* value	FDR	log2(avg diff)	% cells 1	% cells 2
EpiC 6	*Msmo1*	1.64E-38	2.43E-34	0.917	86.70%	54.20%
EpiC 6	*Ivns1abp*	1.66E-38	2.47E-34	0.630	83.30%	47.50%
EpiC 6	*Odc1*	1.75E-38	2.59E-34	1.537	93.80%	67.30%
EpiC 6	*Mgat2*	2.32E-38	3.44E-34	0.553	90.50%	58.00%
EpiC 6	*H2-Eb1*	2.64E-38	3.92E-34	0.503	59.00%	18.10%
EpiC 6	*Cnih1*	4.00E-38	5.93E-34	0.619	95.70%	80.60%
EpiC 6	*Htra1*	7.13E-38	1.06E-33	0.892	94.80%	63.00%
EpiC 6	*Tmem208*	7.15E-38	1.06E-33	0.572	92.90%	76.50%
EpiC 6	*Phlda1*	8.74E-38	1.29E-33	0.704	98.60%	69.70%
EpiC 6	*Atp6ap1*	9.79E-38	1.45E-33	0.623	95.70%	73.40%
EpiC 6	*Cox17*	1.80E-37	2.67E-33	0.623	92.90%	75.00%
EpiC 6	*Stk38l*	3.13E-37	4.64E-33	0.894	70.00%	35.00%
EpiC 6	*Cd24a*	3.17E-37	4.70E-33	0.792	86.70%	51.60%
EpiC 6	*Tmed5*	3.50E-37	5.18E-33	0.702	94.80%	75.70%
EpiC 6	*Anxa3*	4.96E-37	7.34E-33	0.769	92.40%	64.40%
EpiC 6	*Lasp1*	6.08E-37	9.00E-33	0.562	77.60%	39.60%
EpiC 6	*Atp6v1g1*	8.37E-37	1.24E-32	0.592	98.60%	91.10%
EpiC 6	*Gpx8*	1.19E-36	1.76E-32	0.607	73.80%	37.30%
EpiC 6	*Canx*	1.29E-36	1.92E-32	0.661	97.60%	92.50%
EpiC 6	*1810011O10Rik*	1.89E-36	2.80E-32	0.745	93.30%	57.70%
EpiC 6	*Sri*	2.66E-36	3.93E-32	0.652	95.20%	77.50%
EpiC 6	*Tmbim6*	3.34E-36	4.95E-32	0.605	98.60%	94.20%
EpiC 6	*Cnih4*	5.18E-36	7.68E-32	0.686	96.20%	85.20%
EpiC 6	*Slc4a7*	5.48E-36	8.12E-32	0.730	85.20%	48.00%
EpiC 6	*Stt3a*	6.01E-36	8.91E-32	0.561	93.80%	66.10%
EpiC 6	*Ero1l*	6.71E-36	9.94E-32	0.518	85.20%	43.30%
EpiC 6	*9530068E07Rik*	8.46E-36	1.25E-31	0.548	92.40%	68.00%
EpiC 6	*Myo1b*	9.81E-36	1.45E-31	0.600	85.70%	49.50%
EpiC 6	*Maged1*	9.97E-36	1.48E-31	0.579	87.60%	53.50%
EpiC 6	*Laptm4a*	1.31E-35	1.94E-31	0.567	99.00%	97.00%
EpiC 6	*Mmd*	1.51E-35	2.24E-31	0.557	64.30%	26.60%
EpiC 6	*Scd2*	2.62E-35	3.88E-31	0.932	85.20%	49.20%
EpiC 6	*Atp6v0c*	5.37E-35	7.95E-31	0.545	99.50%	96.80%
EpiC 6	*Srsf9*	7.03E-35	1.04E-30	0.515	85.20%	53.70%
EpiC 6	*Spcs3*	7.82E-35	1.16E-30	0.580	82.40%	47.20%
EpiC 6	*Gnai2*	8.50E-35	1.26E-30	0.591	95.20%	79.60%
EpiC 6	*5730559C18Rik*	1.25E-34	1.85E-30	0.506	77.60%	40.20%
EpiC 6	*Ost4*	1.29E-34	1.91E-30	0.553	99.00%	93.70%
EpiC 6	*Phlda3*	1.92E-34	2.84E-30	0.704	99.00%	88.70%
EpiC 6	*Zdhhc20*	2.96E-34	4.39E-30	0.554	90.00%	58.00%
EpiC 6	*Emc7*	3.96E-34	5.87E-30	0.535	94.80%	77.60%

Cluster	Genes	*P* value	FDR	log2(avg diff)	% cells 1	% cells 2
EpiC 6	*Ube2m*	4.69E-34	6.95E-30	0.531	84.30%	54.70%
EpiC 6	*Txndc5*	8.86E-34	1.31E-29	0.510	89.00%	59.50%
EpiC 6	*Myh9*	1.19E-33	1.76E-29	0.851	97.10%	85.10%
EpiC 6	*Acta2*	3.07E-33	4.54E-29	1.658	57.60%	22.30%
EpiC 6	*Sft2d1*	3.90E-33	5.78E-29	0.543	86.20%	57.80%
EpiC 6	*Selk*	4.38E-33	6.49E-29	0.623	98.10%	93.50%
EpiC 6	*Myl6*	4.46E-33	6.60E-29	0.539	100.00%	98.70%
EpiC 6	*Cast*	4.81E-33	7.13E-29	0.593	97.10%	74.40%
EpiC 6	*Hbegf*	6.16E-33	9.13E-29	1.000	85.20%	46.90%
EpiC 6	*Fam167a*	8.02E-33	1.19E-28	0.492	72.90%	32.90%
EpiC 6	*Actb*	9.93E-33	1.47E-28	0.849	100.00%	99.80%
EpiC 6	*Bri3bp*	1.09E-32	1.62E-28	0.546	86.70%	58.60%
EpiC 6	*Pdia6*	1.10E-32	1.63E-28	0.735	97.60%	90.80%
EpiC 6	*Serinc2*	1.25E-32	1.86E-28	0.601	93.30%	77.10%
EpiC 6	*Bnip2*	1.27E-32	1.88E-28	0.554	95.20%	75.90%
EpiC 6	*Tpm2*	1.32E-32	1.96E-28	0.683	98.10%	93.00%
EpiC 6	*Tusc3*	1.61E-32	2.38E-28	0.504	89.00%	58.30%
EpiC 6	*Tpm4*	1.67E-32	2.47E-28	0.657	99.50%	89.60%
EpiC 6	*Ier3ip1*	1.77E-32	2.62E-28	0.522	97.10%	86.20%
EpiC 6	*Pkm*	2.37E-32	3.51E-28	0.530	100.00%	99.50%
EpiC 6	*Rpn2*	3.95E-32	5.86E-28	0.569	97.10%	86.40%
EpiC 6	*Degs1*	4.22E-32	6.26E-28	0.657	96.20%	83.80%
EpiC 6	*Ddost*	5.40E-32	8.00E-28	0.551	96.20%	88.40%
EpiC 6	*Tgfbi*	7.10E-32	1.05E-27	1.034	92.40%	77.80%
EpiC 6	*Cav2*	7.24E-32	1.07E-27	0.505	87.60%	50.40%
EpiC 6	*Gapdh*	7.27E-32	1.08E-27	0.528	100.00%	99.90%
EpiC 6	*Cd44*	7.47E-32	1.11E-27	0.674	99.00%	89.30%
EpiC 6	*Rhob*	1.56E-31	2.31E-27	0.740	94.30%	72.40%
EpiC 6	*Ilk*	1.59E-31	2.36E-27	0.522	86.20%	59.10%
EpiC 6	*Psmc6*	2.72E-31	4.04E-27	0.541	94.80%	83.90%
EpiC 6	*43715*	2.92E-31	4.33E-27	0.585	95.70%	83.50%
EpiC 6	*Ywhag*	3.04E-31	4.51E-27	0.618	96.20%	84.00%
EpiC 6	*Gng5*	3.07E-31	4.55E-27	0.497	100.00%	98.30%
EpiC 6	*Atp6v0e*	3.27E-31	4.85E-27	0.529	98.10%	96.10%
EpiC 6	*Selt*	3.63E-31	5.38E-27	0.565	94.80%	68.00%
EpiC 6	*Tmem33*	4.66E-31	6.90E-27	0.574	92.40%	67.20%
EpiC 6	*Sox9*	5.56E-31	8.24E-27	0.584	79.50%	38.20%
EpiC 6	*Srp19*	6.39E-31	9.46E-27	0.497	95.70%	83.60%
EpiC 6	*4631405K08Rik*	6.95E-31	1.03E-26	0.657	84.30%	47.00%
EpiC 6	*Tceb1*	9.50E-31	1.41E-26	0.541	98.10%	91.10%
EpiC 6	*Fermt1*	1.46E-30	2.16E-26	0.662	88.60%	68.80%

Cluster	Genes	*P* value	FDR	log2(avg diff)	% cells 1	% cells 2
EpiC 6	*Tmem30a*	2.28E-30	3.37E-26	0.559	93.30%	72.10%
EpiC 6	*Tmem128*	2.35E-30	3.48E-26	0.506	92.90%	65.80%
EpiC 6	*Hspa5*	2.85E-30	4.22E-26	0.811	99.50%	94.20%
EpiC 6	*Gpx4*	3.81E-30	5.64E-26	0.542	97.60%	93.60%
EpiC 6	*Ptpn1*	4.84E-30	7.17E-26	0.551	92.90%	74.60%
EpiC 6	*Chmp5*	6.52E-30	9.66E-26	0.506	95.70%	78.10%
EpiC 6	*Anxa8*	9.16E-30	1.36E-25	0.658	97.10%	85.10%
EpiC 6	*Prdx4*	1.04E-29	1.54E-25	0.662	81.90%	51.40%
EpiC 6	*Sec61a1*	1.24E-29	1.83E-25	0.590	93.80%	75.50%
EpiC 6	*Tm9sf3*	4.45E-29	6.59E-25	0.496	93.30%	72.90%
EpiC 6	*Ctsb*	9.41E-29	1.39E-24	0.597	99.00%	95.20%
EpiC 6	*Anxa2*	9.99E-29	1.48E-24	0.507	99.50%	98.40%
EpiC 6	*Slc16a3*	1.30E-28	1.93E-24	0.564	55.70%	20.50%
EpiC 6	*Thbs1*	1.83E-28	2.71E-24	0.684	55.20%	21.30%
EpiC 6	*H13*	3.68E-28	5.45E-24	0.581	92.90%	76.90%
EpiC 6	*Alg5*	5.45E-28	8.07E-24	0.488	81.90%	55.30%
EpiC 6	*Magt1*	1.35E-27	1.99E-23	0.550	82.90%	54.10%
EpiC 6	*Tram1*	1.55E-27	2.30E-23	0.553	86.20%	62.60%
EpiC 6	*Myadm*	3.70E-27	5.48E-23	0.538	79.50%	42.80%
EpiC 6	*Ssr4*	3.95E-27	5.86E-23	0.565	98.60%	94.60%
EpiC 6	*Palld*	8.81E-27	1.30E-22	0.576	93.80%	69.70%
EpiC 6	*Ldha*	1.04E-26	1.53E-22	0.508	100.00%	99.20%
EpiC 6	*Nisch*	1.59E-26	2.35E-22	0.500	89.50%	62.50%
EpiC 6	*Tln1*	1.82E-26	2.70E-22	0.500	85.20%	55.00%
EpiC 6	*Bcap31*	3.03E-26	4.49E-22	0.504	94.80%	88.30%
EpiC 6	*Sigmar1*	4.71E-26	6.98E-22	0.510	87.60%	61.90%
EpiC 6	*Col17a1*	5.09E-26	7.54E-22	0.767	98.10%	94.10%
EpiC 6	*Asun*	6.97E-26	1.03E-21	0.629	61.90%	33.90%
EpiC 6	*Cyp51*	7.67E-26	1.14E-21	0.566	75.20%	41.00%
EpiC 6	*Tuba1c*	1.39E-25	2.05E-21	0.716	99.00%	87.90%
EpiC 6	*Tm9sf2*	1.75E-25	2.60E-21	0.521	91.00%	64.40%
EpiC 6	*Lsr*	2.02E-25	3.00E-21	0.505	89.00%	69.40%
EpiC 6	*Mast4*	2.03E-25	3.01E-21	0.548	88.60%	63.10%
EpiC 6	*Vimp*	2.07E-25	3.07E-21	0.572	91.90%	76.30%
EpiC 6	*Serp1*	3.48E-25	5.15E-21	0.521	97.10%	85.80%
EpiC 6	*Atp2a2*	3.75E-25	5.55E-21	0.654	90.00%	72.80%
EpiC 6	*Tmed2*	4.77E-25	7.07E-21	0.547	99.00%	95.90%
EpiC 6	*Aebp2*	8.63E-25	1.28E-20	0.849	78.10%	52.80%
EpiC 6	*Nedd4*	1.11E-24	1.64E-20	0.533	97.60%	90.60%
EpiC 6	*Ly6e*	1.43E-24	2.12E-20	0.756	98.60%	95.50%
EpiC 6	*Tmed9*	2.66E-24	3.95E-20	0.501	96.70%	92.90%

Cluster	Genes	*P* value	FDR	log2(avg diff)	% cells 1	% cells 2
EpiC 6	*Cd151*	2.71E-24	4.01E-20	0.570	95.20%	83.80%
EpiC 6	*Efnb1*	3.27E-24	4.85E-20	0.501	92.90%	77.60%
EpiC 6	*Gjb2*	7.37E-24	1.09E-19	0.670	74.80%	41.50%
EpiC 6	*Fst*	9.92E-24	1.47E-19	0.647	72.40%	36.90%
EpiC 6	*Pdia4*	1.13E-23	1.68E-19	0.568	93.80%	79.40%
EpiC 6	*Cnn2*	2.70E-23	4.00E-19	0.559	85.70%	58.70%
EpiC 6	*Spcs2*	6.11E-23	9.05E-19	0.513	96.70%	87.70%
EpiC 6	*Macf1*	5.15E-22	7.63E-18	0.486	86.20%	55.60%
EpiC 6	*Ppbp*	9.98E-22	1.48E-17	0.800	12.90%	1.40%
EpiC 6	*Tmem59*	7.82E-21	1.16E-16	0.488	96.70%	91.50%
EpiC 6	*Tagln*	8.59E-21	1.27E-16	0.492	13.30%	1.60%
EpiC 6	*Ybx1*	2.54E-20	3.76E-16	0.510	98.60%	97.40%
EpiC 6	*Mrps35*	2.26E-19	3.35E-15	0.803	81.40%	68.00%
EpiC 6	*Ccnd2*	3.86E-19	5.72E-15	0.565	92.90%	72.60%
EpiC 6	*Tubb5*	7.34E-19	1.09E-14	0.538	99.00%	92.40%
EpiC 6	*Plec*	1.76E-18	2.61E-14	0.589	94.80%	81.50%
EpiC 6	*Ptges*	1.50E-17	2.22E-13	0.527	73.80%	49.40%
EpiC 6	*Procr*	2.78E-17	4.11E-13	0.570	95.70%	85.00%
EpiC 6	*S100a4*	4.41E-17	6.53E-13	0.525	38.10%	15.50%
EpiC 6	*Ctsc*	4.64E-16	6.88E-12	0.947	87.10%	71.10%
EpiC 6	*Ccl8*	3.66E-09	5.42E-05	0.510	9.00%	1.90%
EpiC 6	*Car2*	2.12E-08	3.15E-04	0.696	41.90%	25.20%

P value was caculated based on two-sided likelihood-ratio test for single cell gene expression (bimod option from Seurat package); FDR, Boferroni corrected P value; log2(vag diff), change of the average; % cell 1, % of cells expressing the gene in the given custer; % cell 2, % of cells expressing the gene in all other custers

Supplementary Table 2. Summary information of single cell data processing

Dataset	Cell number after filtered	Gene number after filtered	Differential genes used for clustering	Principal components used
Total CD45+ cells	28,701	15,610	1,550	10
Total CD45- cells	36,114	15,596	1,614	10
Epithelial cells	1,756	14,846	2,174	27
Fibroblasts	31,654	14,646	1,247	13
T cells	8,032	12,472	1,222	13
CD4+ T cells	2,635	12,466	1,263	27
CD8+ T cells	3,812	12,054	1,209	16
Myeloid cells	6,649	13,814	1,992	17

Supplementary Table 3. Antibodies used in this study

Antibody	Supplier	Catalog number	Reactivity species	Origin species	Clonality	Clone	Antigen retrieval	Dilution (usage)
Mki67	Abcam	ab16667	Mouse, Rat, Human, Common marmoset	Rabbit	Monoclonal	SP6	pH=9	1:50 (IHC)
Top2a	Abcam	ab52934	Mouse, Rat, Human	Rabbit	Monoclonal	EP1102Y	pH=9	1:8000 (IHC), 1:10000 (IF)
Aldh3a1	Abcam	ab76976	Mouse, Rat, Human	Rabbit	Polyclonal	/	pH=6	1:200 (IHC), 1:600 (IF)
Atf3	Abcam	ab216569	Mouse, Rat, Human	Rabbit	Polyclonal	/	pH=6	1:200 (IHC), 1:600 (IF)
S100a8	Abcam	ab92331	Mouse, Human	Rabbit	Monoclonal	EPR3554	pH=6	1:500 (IHC), 1:1500 (IF)
Mmp14	Abcam	ab51074	Mouse, Rat, Human	Rabbit	Monoclonal	EP1264Y	pH=9	1:2000 (IHC), 1:6000 (IF)
Itga6	Abcam	ab181551	Mouse, Rat, Human	Rabbit	Monoclonal	EPR18124	pH=9	1:250 (IHC), 1:750 (IF)
CD45	BD	553080	Mouse	Rat	Monoclonal	30-F11 (RUO)	/	1:20 (Flow cytometry)
