# Supplementary material for: Single-cell transcriptomic analysis in a mouse model deciphers cell transition states in the multistep development of esophageal cancer
Source: Nat Commun. 2020 Jul 24;11:3715. doi: 10.1038/s41467-020-17492-y (PMC7381637; doi:10.1038/s41467-020-17492-y)
Supplement: Supplementary file 3 — Reporting Summary [file 41467_2020_17492_MOESM3_ESM.pdf]

nature research

Corresponding author(s): Jianbin Wang, Chen Wu, Dongxin Lin

Last updated by author(s): Jun 18, 2020

Reporting Summary

Nature Research wishes to improve the reproducibility of the work that we publish. This form provides structure for consistency and transparency in reporting. For further information on Nature Research policies, see our Editorial Policies and the Editorial Policy Checklist.

Statistics

For all statistical analyses, confirm that the following items are present in the figure legend, table legend, main text, or Methods section.

n/a	Confirmed	
☐	☒	The exact sample size (*n*) for each experimental group/condition, given as a discrete number and unit of measurement
☐	☒	A statement on whether measurements were taken from distinct samples or whether the same sample was measured repeatedly
☐	☒	The statistical test(s) used AND whether they are one- or two-sided<br>*Only common tests should be described solely by name; describe more complex techniques in the Methods section.*
☒	☐	A description of all covariates tested
☒	☐	A description of any assumptions or corrections, such as tests of normality and adjustment for multiple comparisons
☐	☒	A full description of the statistical parameters including central tendency (e.g. means) or other basic estimates (e.g. regression coefficient) AND variation (e.g. standard deviation) or associated estimates of uncertainty (e.g. confidence intervals)
☐	☒	For null hypothesis testing, the test statistic (e.g. *F*, *t*, *r*) with confidence intervals, effect sizes, degrees of freedom and *P* value noted<br>*Give P values as exact values whenever suitable.*
☒	☐	For Bayesian analysis, information on the choice of priors and Markov chain Monte Carlo settings
☐	☒	For hierarchical and complex designs, identification of the appropriate level for tests and full reporting of outcomes
☐	☒	Estimates of effect sizes (e.g. Cohen's *d*, Pearson's *r*), indicating how they were calculated

Our web collection on statistics for biologists contains articles on many of the points above.

Software and code

Policy information about availability of computer code

Data collection	CellRanger (v2.1.0)
Data analysis	R (v 3.5.1) with packages Seurat (v 2.3.4), GSVA (v 1.30.0), DESeq2 (v 1.22.2), Limma(v 3.38.3), monocle2 (v 2.10.1), SCENIC (v 1.1.0)<br>HISAT2 (v 2.1.0)<br>HTSeq (v 0.6.1p1)<br>CIBERSORT (v 1.06)<br>CellPhoneDB (v 2.0.6)<br>GraphPad Prism (v 7.0.4)<br>inForm (v 2.4.2)<br>BD FACSDiva (v 8.0.1)<br>Example scripts to process and analyze data is available at https://github.com/ESCCemAll/scESCC_mice, detailed information will be available from the corresponding author upon reasonable request.

For manuscripts utilizing custom algorithms or software that are central to the research but not yet described in published literature, software must be made available to editors and reviewers. We strongly encourage code deposition in a community repository (e.g. GitHub). See the Nature Research guidelines for submitting code & software for further information.

Data

Policy information about availability of data

All manuscripts must include a data availability statement. This statement should provide the following information, where applicable:
- Accession codes, unique identifiers, or web links for publicly available datasets
- A list of figures that have associated raw data
- A description of any restrictions on data availability

The raw sequencing data and processed gene expression matrix of mouse model have been deposited in GSA (Genome Sequence Archive in BIG Data Center, Beijing Institute of Genomics, Chinese Academy of Sciences, http://gsa.big.ac.cn) under the accession number CRA002118. The raw sequencing data of human esophageal tissues has been deposited in GSA-Human (https://bigd.big.ac.cn/gsa-human) under the accession number HRA000093. The Metadata for sequenced samples and the Source Data underlying Figs.1b, c, 2a–d, f, 3a–f, 4a–d, 5a–d, 6a–c, 7a–c and Supplementary Figs. 1c, 2b, c, 3a–d, f, g, 4b, 5c, d, 6b, d, e and 7b are provided as a Source Data file. All the other data supporting the findings of this study are available within the article and its supplementary information files and from the corresponding author upon reasonable request. A reporting summary for this article is available as a Supplementary Information file.

Field-specific reporting

Please select the one below that is the best fit for your research. If you are not sure, read the appropriate sections before making your selection.

☒ Life sciences ☐ Behavioural & social sciences ☐ Ecological, evolutionary & environmental sciences

For a reference copy of the document with all sections, see nature.com/documents/nr-reporting-summary-flat.pdf

Life sciences study design

All studies must disclose on these points even when the disclosure is negative.

Sample size	No statistical methods were used to pre-determine sample size. For mouse single cell assays, due to the low number of epithelial cells, the numbers of mouse esophagi were determined to obtain as many epithelial cells as possible but restricted by the total cell number limitation of single 10x assay. As a total, we analyzed the transcriptomes of 66,089 cells. Selection and filtering were performed as demonstrated in method section. For human samples, sample size is restricted due to practical constraints of patient Recruitment and sample collection.
Data exclusions	This exclusion criteria were pre-established and generally used in 10x single cell assays. Cells expressing more than 10% of mitochondrial genes, less than 500 total genes were excluded.
Replication	Transcriptome data from 66089 single cells, obtained from at least 17 mice of each pathology stage to reduce bias caused by individual. For Immunohistochemistry and immunofluorescent analysis, similar staining results must be observed in over 3 visual fields, and statistic results were provided where available.
Randomization	Mice were randomly allocated in six groups in our study. Human samples were not randomized due to patient recruitment and sample collection based on pathology stage.
Blinding	Blinding was not appropriate in this study. The mice was sacrificed at a carcinogen-treatment time order. For human samples, groups are allocated based on pathology stage not the investigators.

Reporting for specific materials, systems and methods

We require information from authors about some types of materials, experimental systems and methods used in many studies. Here, indicate whether each material, system or method listed is relevant to your study. If you are not sure if a list item applies to your research, read the appropriate section before selecting a response.

Materials & experimental systems

n/a	Involved in the study	
☐	☒	Antibodies
☒	☐	Eukaryotic cell lines
☒	☐	Palaeontology and archaeology
☐	☒	Animals and other organisms
☐	☒	Human research participants
☒	☐	Clinical data
☒	☐	Dual use research of concern

Methods

n/a	Involved in the study	
☒	☐	ChIP-seq
☐	☒	Flow cytometry
☒	☐	MRI-based neuroimaging

Antibodies

Antibodies used	Antibody used for flow cytometry:

1; CD45-FITC (BD; 553080; Rat anti Mouse; monoclonal[30-F11]; dilution: 1:20; https://www.bdbiosciences.com/us/applications/research/stem-cell-research/cancer-research/mouse/fitc-rat-anti-mouse-cd45-30-f11/p/553080)
Antibodies used for immunohistochemistry (IHC) and immunofluorescent (IF) staining:
1; Mki67(Abcam; ab16667; Rabbit anti Mouse, Rat, Human, Common marmoset; monoclonal [SP6]; dilution: 1:50 for IHC; https://www.abcam.com/ki67-antibody-sp6-ab16667.html)
2; Top2a (Abcam; ab52934; Rabbit anti Mouse, Rat, Human; monoclonal [EP1102Y]; dilution: 1:8000 for IHC, 1:10000 for IF; https://www.abcam.cn/topoisomerase-ii-alpha-antibody-ep1102y-ab52934.html)
3; Aldh3a1 (Abcam; ab76976; Rabbit anti Mouse, Rat, Human; polyclonal; dilution: 1:200 for IHC, 1:600 for IF; https://www.abcam.cn/aldh3a1-antibody-ab76976.html)
4; Atf3 (Abcam; ab216569; Rabbit anti Mouse, Rat, Human; polyclonal; dilution: 1:200 for IHC, 1:600 for IF; https://www.abcam.com/atf3-antibody-ab216569.html)
5; S100a8 (Abcam; ab92331; Rabbit anti Mouse, Human; monoclonal [EPR3554]; dilution: 1:500 for IHC, 1:1500 for IF; https://www.abcam.com/mrp8-antibody-epr3554-ab92331.html)
6; Mmp14 (Abcam; ab51074; Rabbit anti Mouse, Rat, Human; monoclonal [EP1264Y]; dilution: 1:2000 for IHC, 1:6000 for IF; https://www.abcam.com/mmp14-antibody-ep1264y-ab51074.html)
7; Itga6 (Abcam; ab181551; Rabbit anti Mouse, Rat, Human; monoclonal [EPR18124]; dilution: 1:250 for IHC, 1:750 for IF; https://www.abcam.com/integrin-alpha-6-antibody-epr18124-ab181551.html)

Validation: All primary antibodies are tested and characterized as specific in human tissues, by the manufacturers, and are widely cited. Antibody-specific validations are available as indicated on the manufacturers' web page. Links are listed in the "Antibodies Used" section above.

Animals and other organisms

Policy information about studies involving animals; ARRIVE guidelines recommended for reporting animal research

Laboratory animals: Eight-week-old female C57BL/6 mice, housing conditions and treatment details were provided in method section.

Wild animals: This study didn't involve wild animals.

Field-collected samples: This study didn't involve field-collected samples.

Ethics oversight: Animal experiments in this study were conducted in compliance with approved protocols and guidelines from the Institutional Animal Care and Use Committee of the Chinese Academy of Medical Sciences.

Note that full information on the approval of the study protocol must also be provided in the manuscript.

Human research participants

Policy information about studies involving human research participants

Population characteristics: Patients received no chemotherapy or radiotherapy before endoscopy or surgery. Age and gender of the patients weren't restricted. The number of patients are 88; age ranged from 35 to 80 (median 66); 22 females and 66 males; pathology stage range from inflammation to invasive carcinoma.

Recruitment: Human samples were randomly collected during surgery in Linzhou Esophageal Cancer Hospital (Henan Province, China) between 2016 and 2019.

Ethics oversight: This study was approved by the Institutional Review Boards of Cancer Hospital, Chinese Academy of Medical Sciences and informed consent was obtained from each patient.

Note that full information on the approval of the study protocol must also be provided in the manuscript.

Flow Cytometry

Plots

Confirm that:

- [x] The axis labels state the marker and fluorochrome used (e.g. CD4-FITC).
- [x] The axis scales are clearly visible. Include numbers along axes only for bottom left plot of group (a 'group' is an analysis of identical markers).
- [x] All plots are contour plots with outliers or pseudocolor plots.
- [x] A numerical value for number of cells or percentage (with statistics) is provided.

Methodology

Sample preparation: Cells from mouse esophagus were collected, details provided in method section.

Instrument: FACS Aria (BD Biosciences)

Software	BD FACSDiva (v 8.0.1)
Cell population abundance	We determined the live cell counts using hemocytometer after sorting, and the cell concentration was adjusted to over 300 cells/μL before 10x assays.
Gating strategy	The FSC/SSC gating strategy was used to exclude cell debris and doublets. CD45-FITC antibody staining was used as described in method section.

[x] Tick this box to confirm that a figure exemplifying the gating strategy is provided in the Supplementary Information.
